# Recent Advances in Enantioselective Photochemical Reactions of Stabilized Diazo Compounds

**DOI:** 10.3390/molecules24173191

**Published:** 2019-09-02

**Authors:** Ting-Bi Hua, Qing-Qing Yang, You-Quan Zou

**Affiliations:** College of Materials and Chemical Engineering, Key Laboratory of Inorganic Nonmetallic Crystalline and Energy Conversion Materials, China Three Gorges University, 8 Daxue Road, Yichang 443002, Hubei, China

**Keywords:** diazo compounds, enantioselectivity, photocatalysis, Wolff rearrangement

## Abstract

Diazo compounds have proven to be a useful class of carbenes or metal carbenoids sources under thermal, photochemical, or metal-catalyzed conditions, which can subsequently undergo a wide range of synthetically important transformations. Recently, asymmetric photocatalysis has provoked increasing research interests, and great advances have been made in this discipline towards the synthesis of optically enriched compounds. In this context, the past two decades have been the most productive period in the developments of enantioselective photochemical reactions of diazo compounds due to a better understanding of the reactivities of diazo compounds and the emergence of new catalytic modes, as well as easier access to and treatment of stabilized diazo compounds. This review highlights these impressive achievements according to the reaction type, and the general mechanisms and stereochemical inductions are briefly discussed as well.

## 1. Introduction

**Figure 1:**
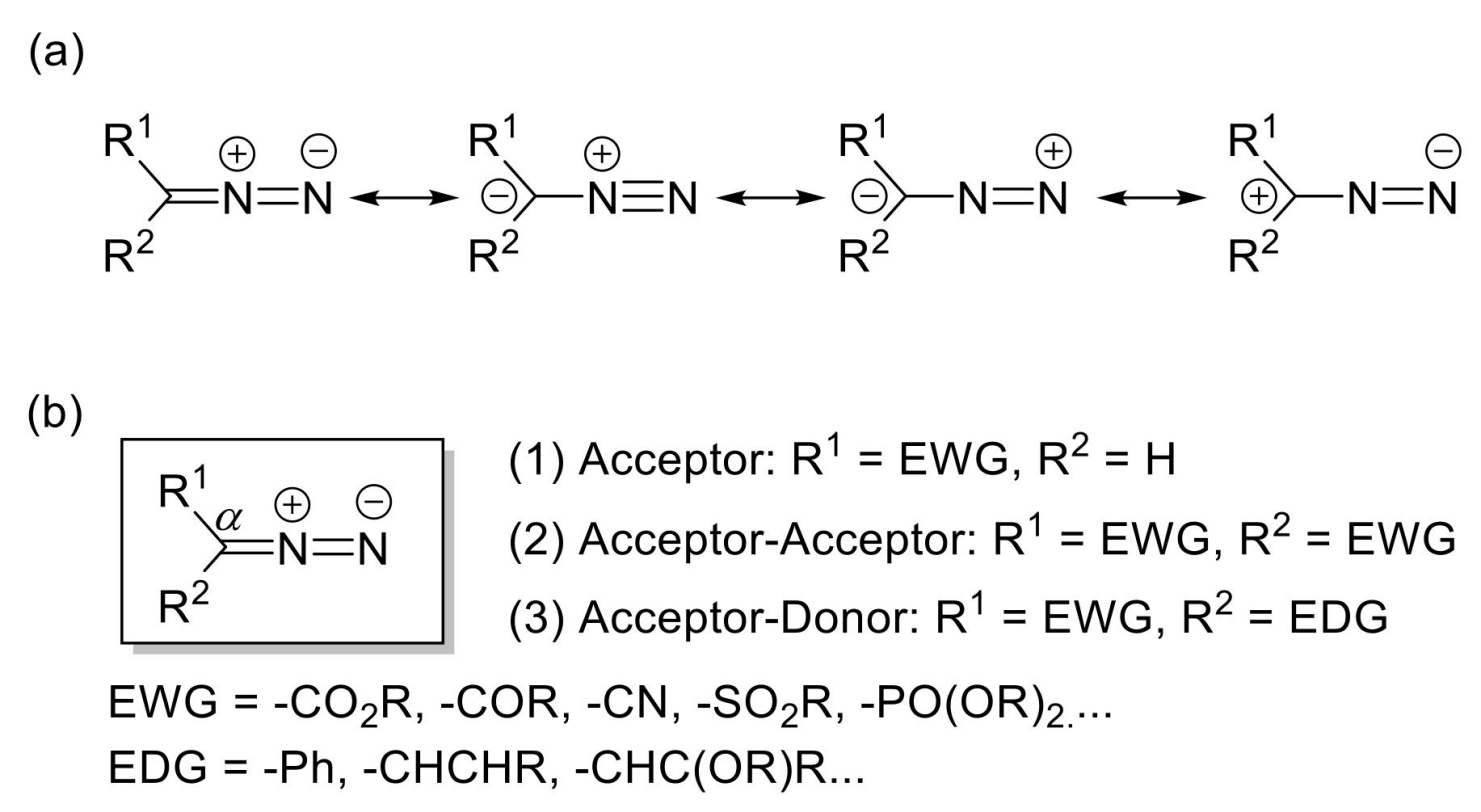
(**a**) Resonance structures of diazo compounds; (**b**) Classification of stabilized diazo compounds.

Diazo compounds are a class of charge neutral organic compounds containing a diazo group bound to a carbon atom, which can resonance into different structures (Figure 1a) [1]. Since its chemistry has existed for more than a century [2], diazo compounds have been rendered to be versatile building blocks and will continue to play an important role in organic synthesis. Generally, their thermal, photoinitiated, or metal-catalyzed decomposition leads to the corresponding carbenes and metal carbenoids, which can subsequently undergo various transformations, including insertion reactions to C-H, O-H, Si-H, N-H, S-H bonds, cyclopropanation, Wolff rearrangement, addition to forming a ylide with a heteroatom and radical reactions [3,4,5,6,7,8,9,10,11,12,13]. Among various diazo compounds, stabilized diazo compounds are common reaction starting materials owing to their easier preparation and handling, as well as the high reactivity for elaborating into different new molecules [8,14]. Typically, they are classified into three categories (Figure 1b): acceptor (with one EWG group bound to α- carbon atom, EWG: electron-withdrawing group), acceptor-acceptor (with two EWG groups bound to an α-carbon atom), and acceptor-donor (with one EWG group and one EDG group bound to α-carbon atom, EDG: electron-donating group) substituted diazo compounds [15,16].

One of the major goals for the advancement of modern organic synthesis is the development of green and efficient methods that allow the rapid construction of chiral molecules from readily available starting materials [17]. In this endeavor, photocatalysis represents a unique and green strategy that has contributed much to the developments towards the synthesis of optically enriched compounds [18,19,20,21,22,23,24] and has also renewed interest in the asymmetric photochemistry of diazo compounds. The past two decades have been the most productive period of this chemistry, which is achieved by a better understanding of the reactivity of diazo compounds and the emergence of new catalytic modes. Despite the two recent elegant reviews by McKervey [8] and Gryko [11] covering on the use of diazo compounds in photochemistry, in this review, we will critically focus on the most recent impressive work in enantioselective photochemical reactions of stabilized diazo compounds from the following three aspects: (1) asymmetric cyclopropanation, (2) asymmetric reactions based on in situ Wolff rearrangement, and (3) asymmetric alkylation. Particular emphasis is also placed on the general mechanisms and stereochemical inductions underpinning these processes.

## 2. Asymmetric Cyclopropanation

### 2.1. Intermolecular Asymmetric Cyclopropanation

The cyclopropane moiety is widely present as a common structural subunit in diverse natural products and biologically active compounds. It can serve as a versatile and important building block in organic synthesis due to its unique combination of reactivity and structural properties [25]. As a result, great research efforts have been devoted to the stereoselective construction of such three-membered carbocyclic rings over the last few decades [26]. Of the most important methods available, asymmetric cyclopropanation of olefins, the typical carbene transfer reaction, is an attractive strategy for the synthesis of optically active cyclopropanes [27]. Chiral metallosalen complexes have been well-recognized to serve as a powerful class of catalysts for enantioselective carbene transfer reactions such as asymmetric cyclopropanation [28,29,30].

**Scheme 1:**
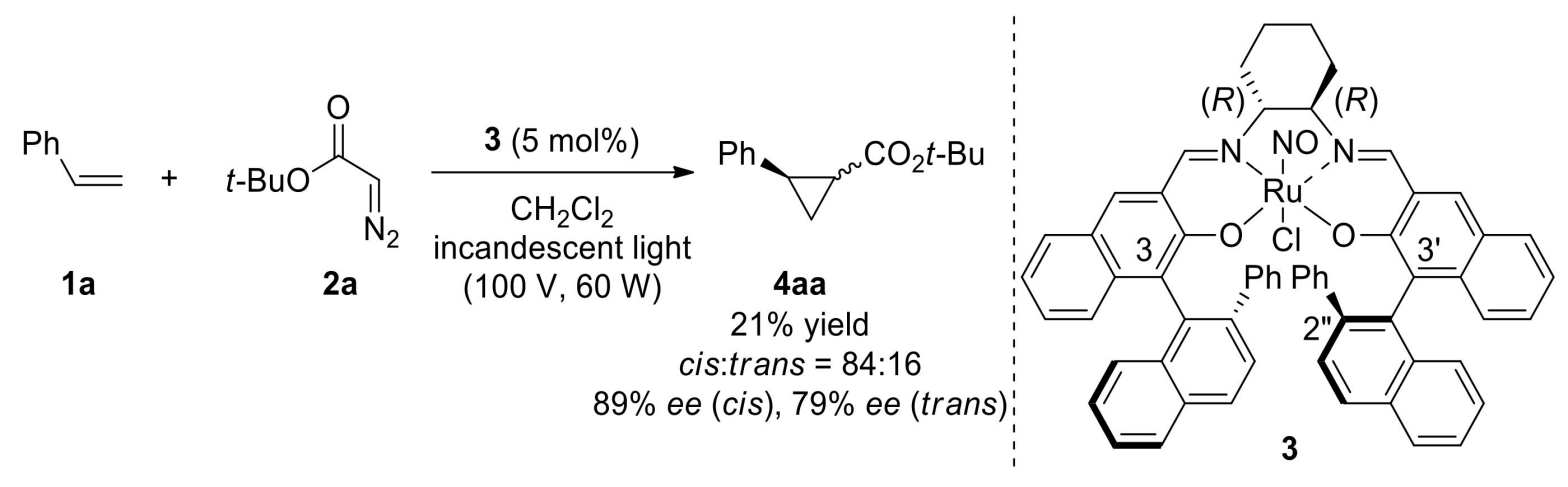
Asymmetric intermolecular cyclopropanation of styrene using the (ON^+^)Ru-salen complex.

The Katsuki group developed a series of highly *cis*- and enantioface-selective cyclopropanation reactions with chiral (ON^+^)Ru-salen complexes as catalysts [31,32,33,34,35]. In 1999, they reported a (*R*,*R*)-(ON^+^)(salen)ruthenium(II) [(*R*,*R*)-Ru-salen] complex catalyzed the unprecedented highly *cis*- and enantioselective cyclopropanation of styrene **1a** (89% ee, *cis*:*trans* = 84:16) when the reaction was carried out under the irradiation of incandescent light (Scheme 1) [31].

**Scheme 2:**
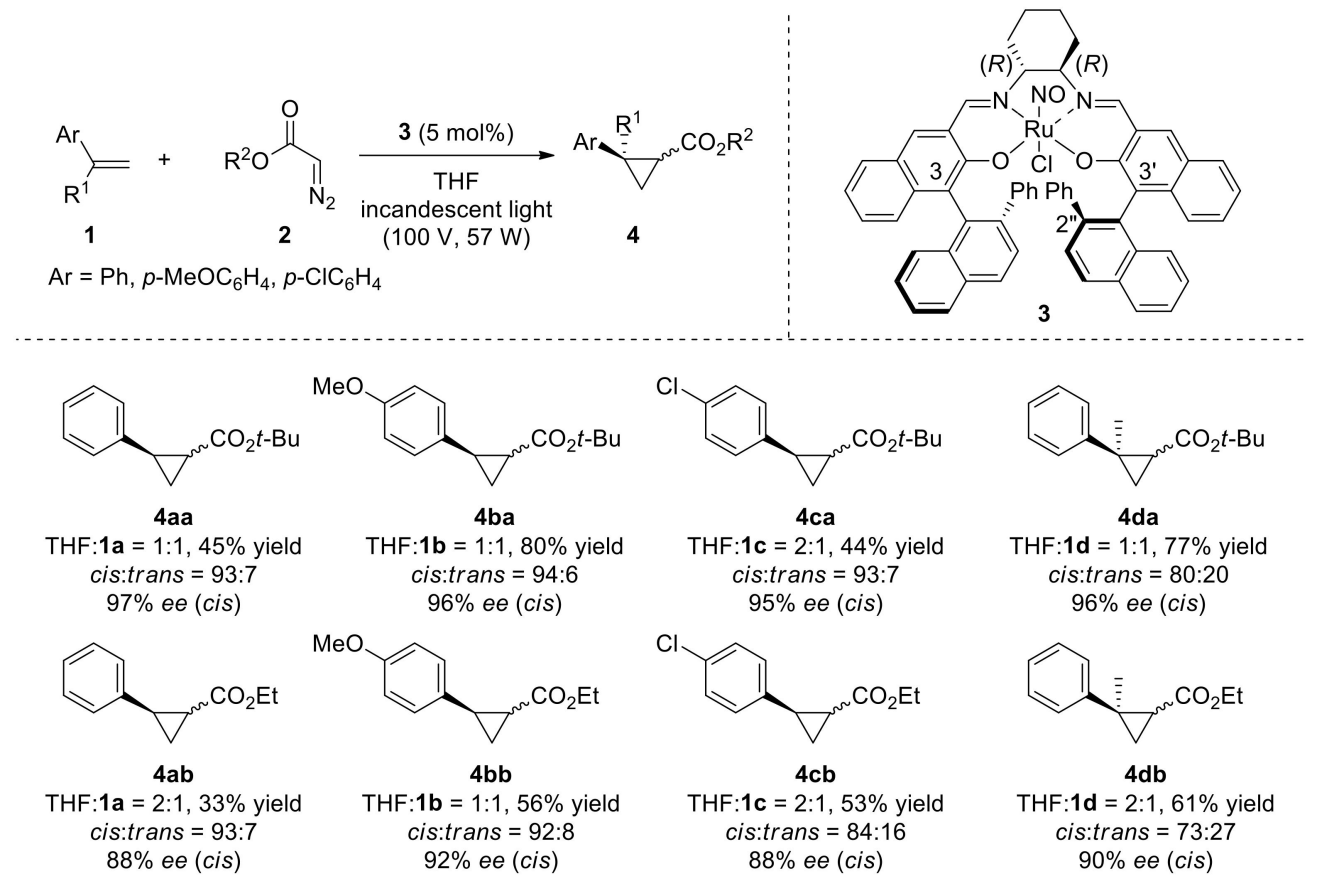
Asymmetric intermolecular cyclopropanation of various olefins and α-diazoacetates (THF: tetrahydrofuran).

However, several challenges remained under these reaction conditions: undesired decomposition of α-diazoacetate and non-catalyzed cyclopropanation, which is non-selective thus diminishes the enantiomeric excess of the desired cyclopropane product. In order to overcome these challenges, different parameters such as the wavelength of the irradiated light, the effect of solvent and substrate concentration were screened by the same group [32,33]. Three different kinds of solvents (i. high dissolving power; ii. high dissolving power and high coordinating ability; iii. poor dissolving power) were examined, and the reversal of enantioselection in the formation of the major *cis*-isomer was observed in the reactions with the solvents of high dissolving power and poor dissolving power. The catalyst was completely dissolved in the solvents of high dissolving power and functioned as a monomeric activated species which decomposed α-diazoacetate upon irradiation, while the catalyst is not dissolved in the solvent with poor dissolving power and the activation should occur on the surface of the insoluble solid catalyst. It was demonstrated that association state (monomeric or aggregated) of the catalyst in different solvents would have an influence on the conformation of the salen ligand and, in turn, the enantioface selection by metallosalen complex since salen ligands are highly pliable. Asymmetric cyclopropanation of various olefins **1** and α-diazoacetates **2** under optimal reaction conditions was performed smoothly, providing the corresponding products **4** with excellent enantioselectivity, as well as high *cis*-selectivity (Scheme 2). For example, various styrenes with an electron-donating group and electron-withdrawing group on the aromatic ring were tolerated in this reaction. α-Diazoacetates of different ester groups also proceeded well, and the observed results suggested that the diminished selectivity was attributed to the decrease of the steric bulkiness of the ester group. Control experiments with various (ON^+^)Ru-salen complexes as catalysts showed that the 2”-substituents on the naphthyl ring of complexes were essential to the stereoselectivities and also supported the assumption that olefins approached the carbenoid-carbon passing by the C3 (3’)-naphthyl ring of the catalysts [33].

### 2.2. Intramolecular Asymmetric Cyclopropanation

**Scheme 3:**
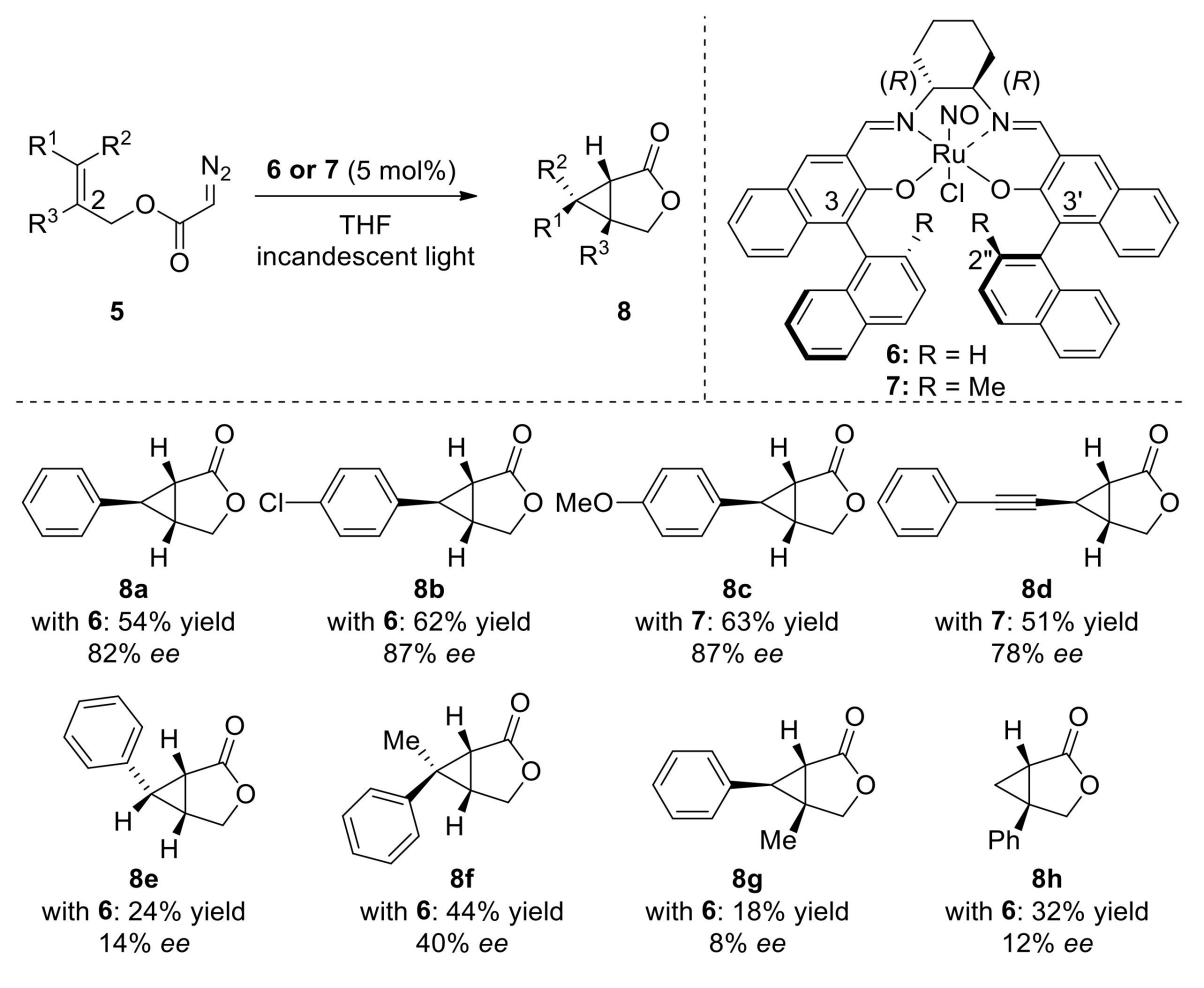
Asymmetric intramolecular cyclopropanation of (*E*)-2-alkenyl α-diazoacetates with (nitroso)(salen)-ruthenium(II) complexes as catalysts.

**Scheme 4:**
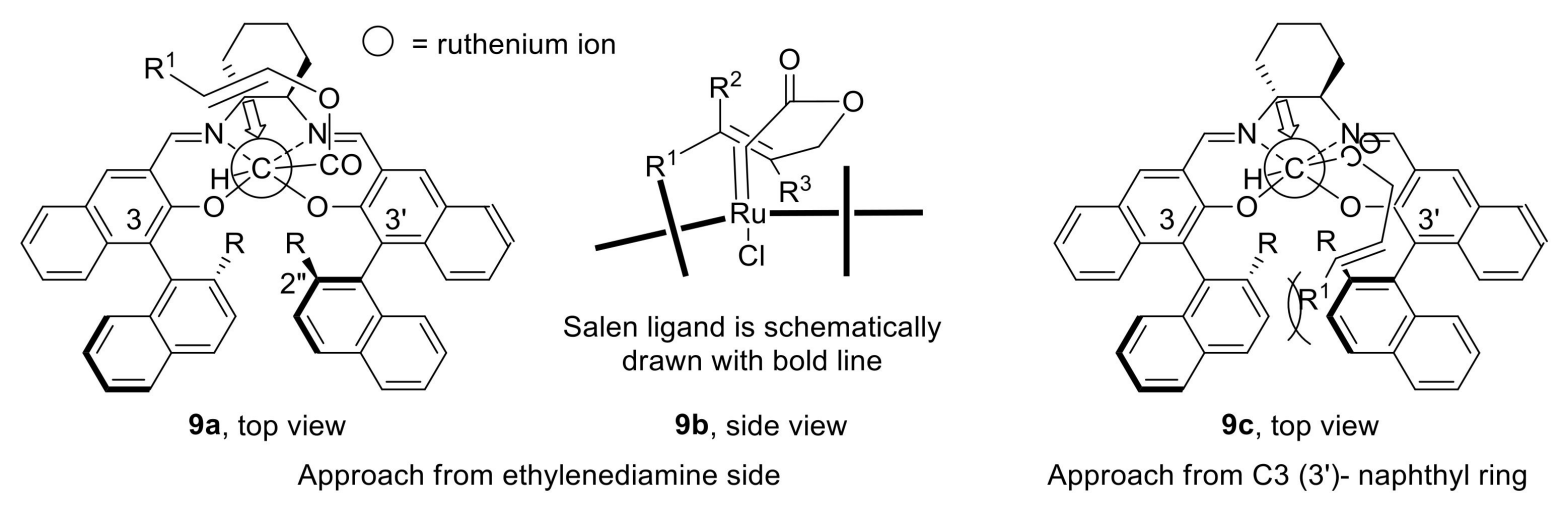
Mechanistic model.

In 2001, Katsuki and co-workers further expanded this catalytic system to intramolecular asymmetric cyclopropanation of (*E*)-2-alkenyl α-diazoacetates **5** using complex **6** or **7** as a catalyst (Scheme 3). Several *trans*-substituted allyl α-diazoacetates, such as different aryl (**8a**–**8c**), alkynyl (**8d**) substituted allyl α-diazoacetates were suitable for the process under photo-irradiated conditions, displaying good reactivity and stereoselectivity. In addition, the introduction of *Z*-substituent (**8e**–**8f**) and 2-substituent (**8g**–**8h**) on the alkene moiety depressed the enantioselectivity. Based on the above results, the authors proposed the mechanistic model (Scheme 4). The olefinic moiety approached with an orientation perpendicular to Ru-C bond from the ethylenediamine side of the catalysts probably due to the steric repulsion with the substituents on the C3 (3’)-naphthyl ring in contrast to the intermolecular cyclopropanation (*cf*. **9a** and **9c**) followed by anti-clockwise rotation (**9b**), thereby giving the desired product. *E*-substituent (R^1^) and *Z*-substituent (R^2^) do not cause severe steric repulsion upon the approach of the olefinic unit due to the distorted stepped conformation of salen ligand, but R^2^ was considered to suffer some hindrance upon the rotation, therefore depression of enantioselectivity to some extent. The introduction of 2-substituent (R^3^) decayed enantioselectivity owing to the destabilization of the orientation [34].

**Scheme 5:**
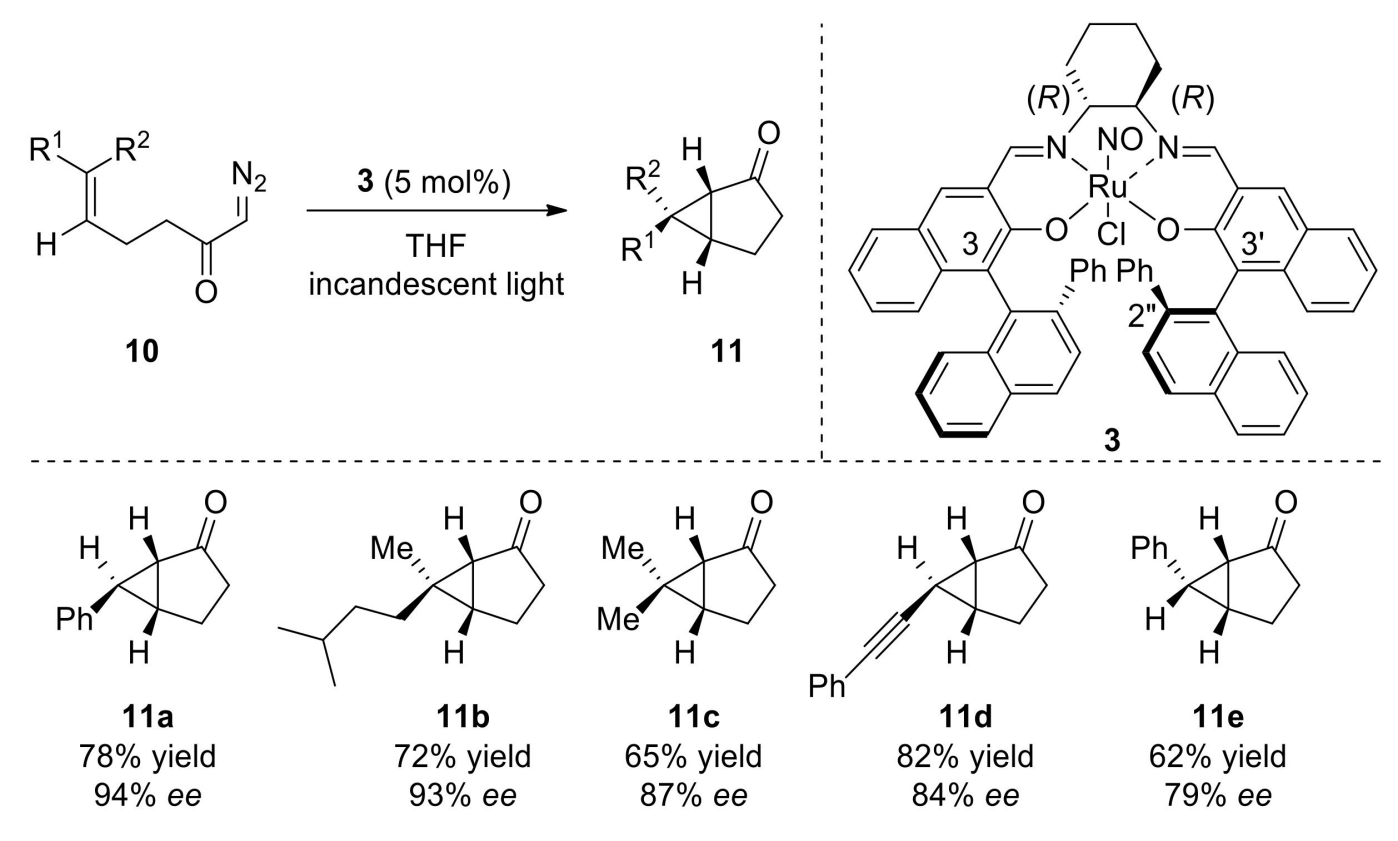
Asymmetric intramolecular cyclopropanation of alkenyl diazo ketones using Ru-salen as a catalyst.

In addition to alkenyl diazoester compounds, the Katsuki group subsequently extended this strategy to intramolecular cyclopropanation of alkenyl diazoketones including trisubstituted alkenyl, alkynyl alkenyl, and *Z*-phenyl alkenyl diazoketones **10** (Scheme 5) [35]. It is worthy of mentioning that alkenyl diazoketones are much more difficult substrates for the stereocontrol in intramolecular cyclopropanation due to the transition state conformation away from the chiral auxiliary [36] and the formation of more reactive carbenoid intermediates than those from diazoesters [37]. Based on the experimental results, the authors assumed that 2”-phenyl substituents effectively regulated the orientation of the incoming alkene moiety of the carbenoid intermediate by protruding towards it. And it was suggested that the approach of the olefin moiety was mainly governed by the chiral center of the diamine part, which had a great influence on the conformation of the salen ligand.

## 3. Asymmetric Reactions Based on In Situ Wolff Rearrangement

**Scheme 6:**
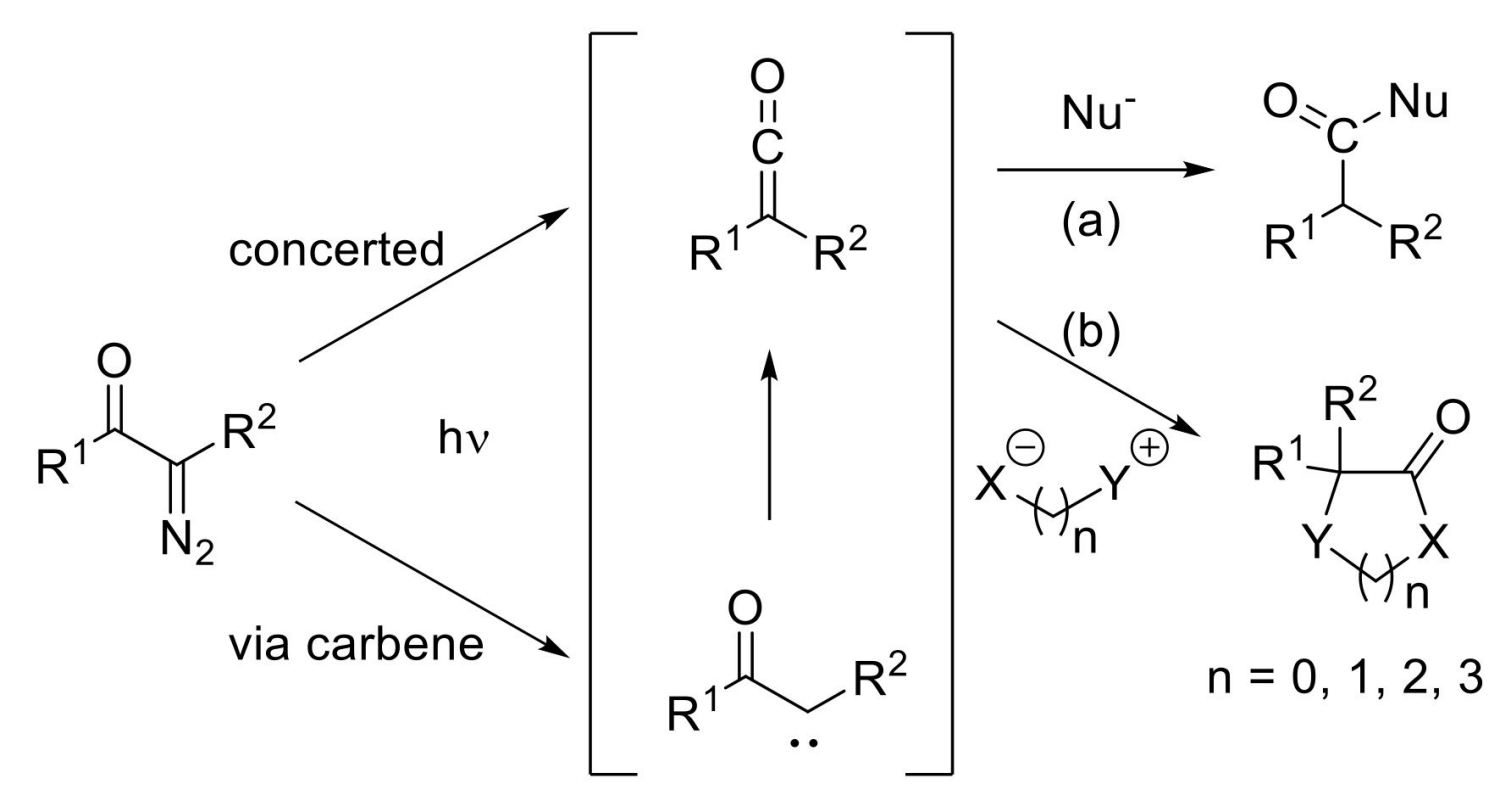
General scheme of the Wolff rearrangement and the subsequent trapping reactions: (**a**) nucleophilic addition; (**b**) cycloaddition reactions.

The Wolff rearrangement, known for over 100 years, represents one of the most useful and widely studied transformations of α-diazocarbonyl compounds for one-carbon (methylene) extension, which has contributed much to the growth of organic synthesis [38]. The photolytic Wolff rearrangement of α-diazocarbonyl compounds is a photochemical process enabling the efficient, traceless synthesis of ketene intermediates via nitrogen elimination and 1,2-rearrangement under mild conditions. The resulting ketene intermediates are not usually isolated but directly trapped with various reactants in the course of the reactions. For instance, it could react with various nucleophiles to provide carboxylic acid derivatives (Scheme 6a), and cycloaddition reactions could take place with some dipoles or unsaturated compounds, affording diversely functionalized cycloadducts (Scheme 6b). In this context, asymmetric reactions based on the in situ Wolff rearrangement, including these two aspects are briefly discussed.

### 3.1. Nucleophilic Addition Based on the In Situ Wolff Rearrangement

#### 3.1.1. Oxygen Nucleophilic Addition

**Scheme 7:**
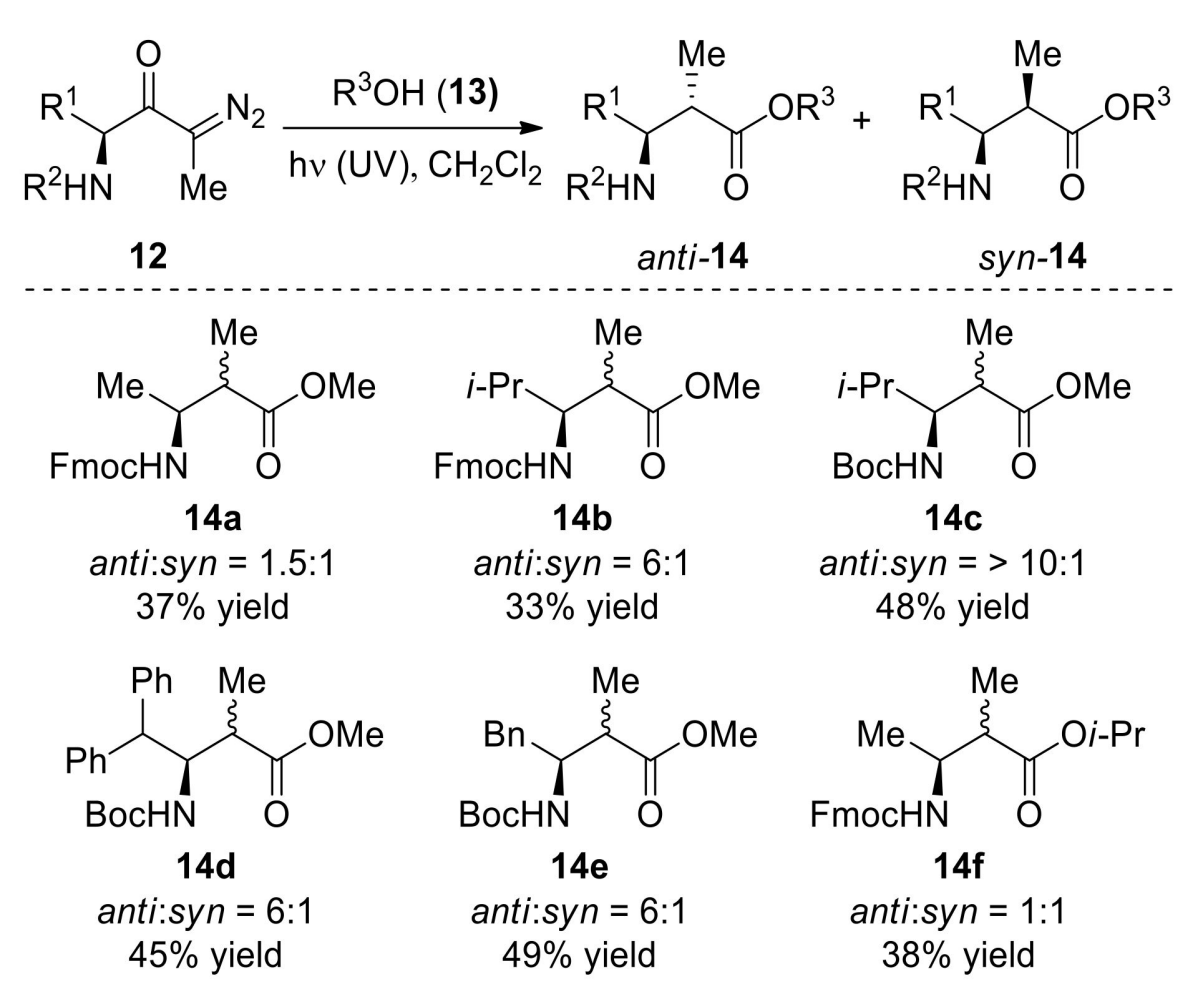
Asymmetric Wolff rearrangement with α-diazoketones. (UV: ultra violet).

**Scheme 8:**
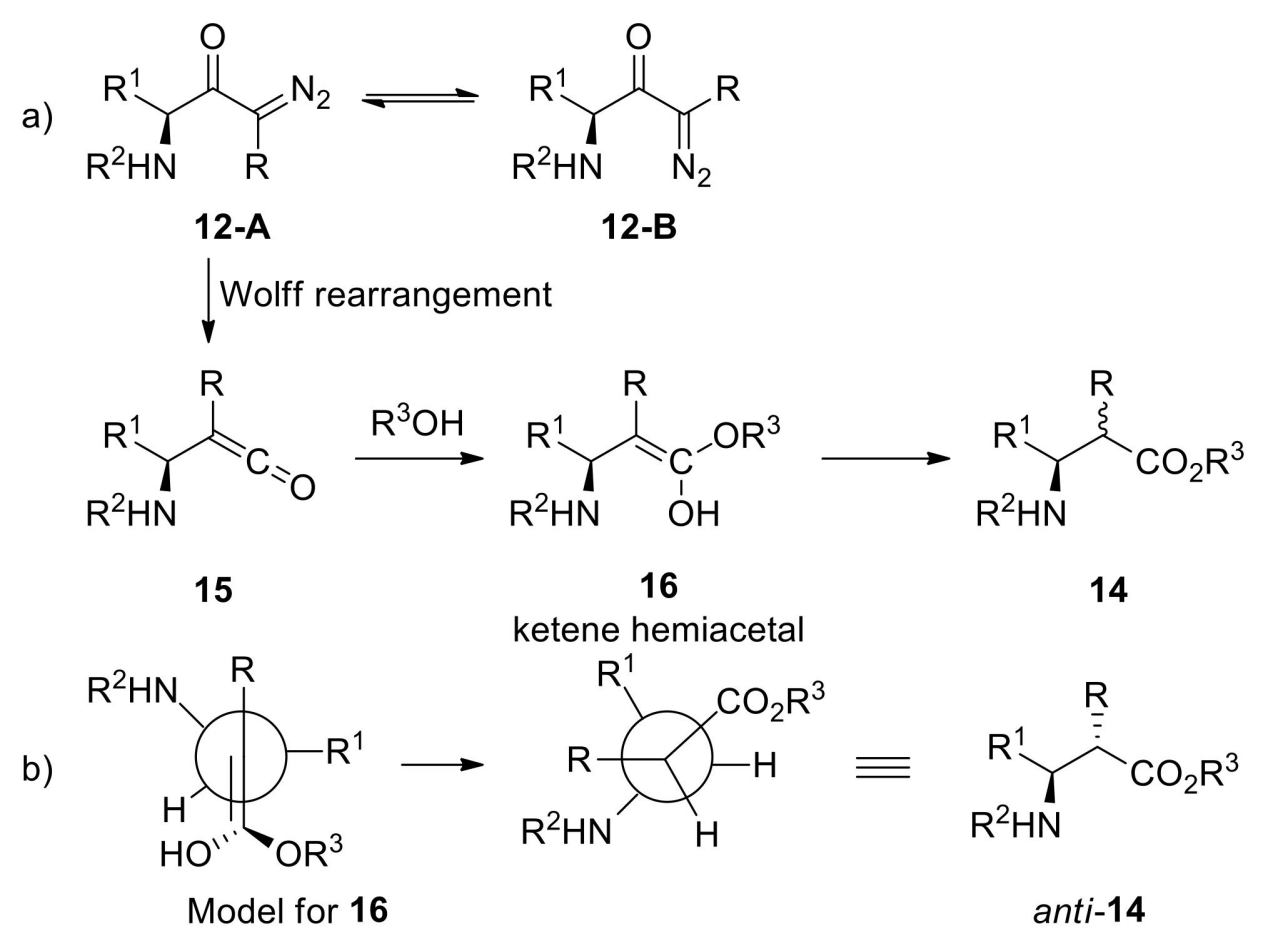
(**a**) Reaction mechanism; (**b**) Stable conformation of the transition state of the tautomerization.

In 2000, Yang et al. developed a photoinduced asymmetric Wolff rearrangement reaction of α-alkylated-α-diazoketones **12** for the stereoselective synthesis of α-substituted-β-amino acid derivatives **14** with good stereoselectivity (Scheme 7) [39]. The reaction proceeded via the capture of ketenes generated from Wolff rearrangement by alcohols and tautomerization of the resulting ketene hemiacetal intermediate (Scheme 8a). Factors that may have a great impact on the stereochemistry were examined, including the temperature dependence and steric effects, which influenced the stereochemistry. The fact that higher yield could be obtained at a higher temperature implies the relative ease of rotation of the diazo group around the C1-C2 bond at higher temperatures. The authors proposed the most stable conformation of the transition state of tautomerization for the resulting ketene hemiacetal intermediate **16** (Scheme 8b). The proton could approach from one side of the ketene hemiacetal due to the release of the strain between R^1^ and R, as well as between R^2^ and R to the maximum extent in the transient state. Therefore, increasing the size of R^1^, R^2^, and R would improve the diastereoselectivity. R^3^ points far away from group R^1^ and R^2^; thus the effect of R^3^ on prochiral center is much less than that of group R^1^ and R^2^. The proposed model is consistent with the experimental results.

**Scheme 9:**
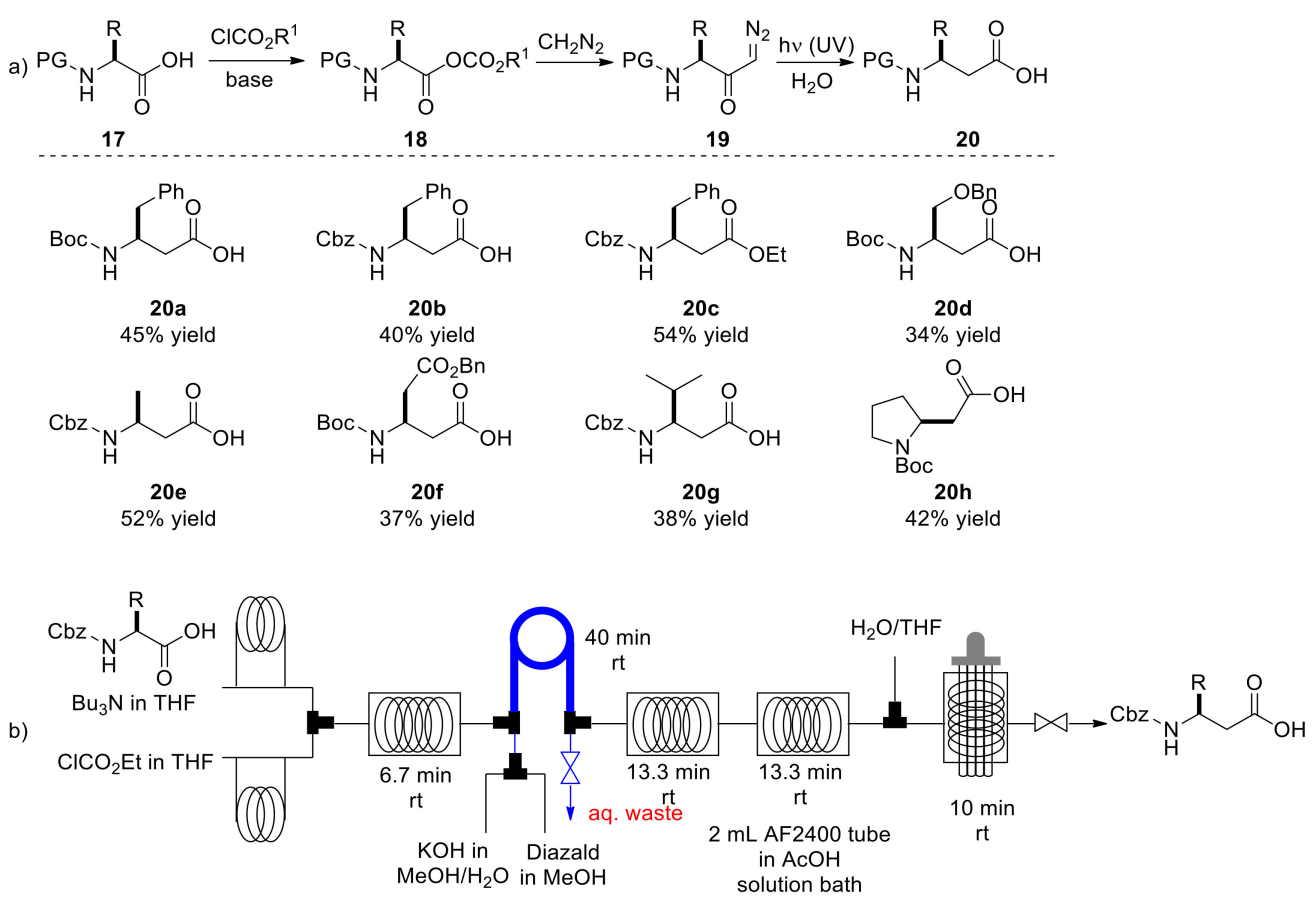
(**a**) Continuous flow synthesis of β-amino acids from protected α-amino acids; (**b**) Flow set-up for the continuous four-step synthesis of β-amino acids from protected α-amino acids. (PG: protecting group, Diazald: N-methyl-N-nitroso-p-toluene-sulfonamide).

In 2014, the Kappe group disclosed a fully continuous flow synthesis of β-amino acids from the respective protected α-amino acid **17**, in which successive reactions have to be accomplished. The photochemical Wolff rearrangement of the diazoketone accompanying with the interception of the ketene intermediate with water completed the final step, providing the β-amino acids **20** in reasonable overall yields (Scheme 9) [40].

**Scheme 10:**
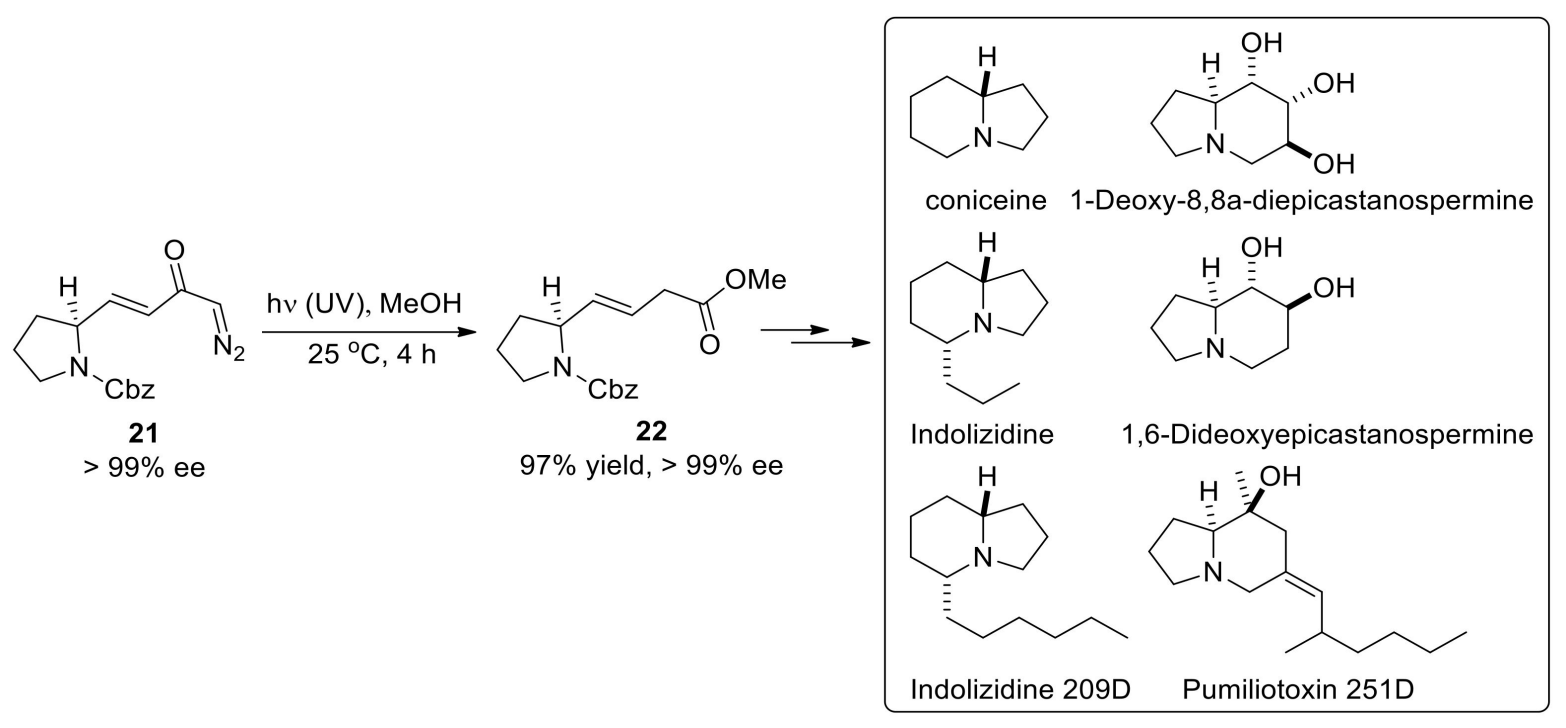
Diversity-oriented synthesis of different types of indolizidines from α,β-unsaturated diazoketones. (The UV light was generated by an Osram 150 Xenon lamp accommodated in an Oriel Model 8500 Universal arc lamp source with focusing quartz lens, a water-filled infrared filter, and a thermostated cell holder.).

This success showed the advantage that the chiral center residing in the molecules could influence the stereochemistry of the newly established chiral center of the Wolff rearrangement product. Several developments have been made towards this trend. For instance, Burtoloso and co-workers demonstrated that α,β-unsaturated diazoketones **21** were powerful platforms to be applied in a number of diverse approaches for the short and diversity-oriented synthesis of indolizidines (Scheme 10) [41,42]. The concise route benefits from the high yielding photochemical Wolff rearrangement of α,β-unsaturated diazoketone under mild reaction conditions.

**Scheme 11:**
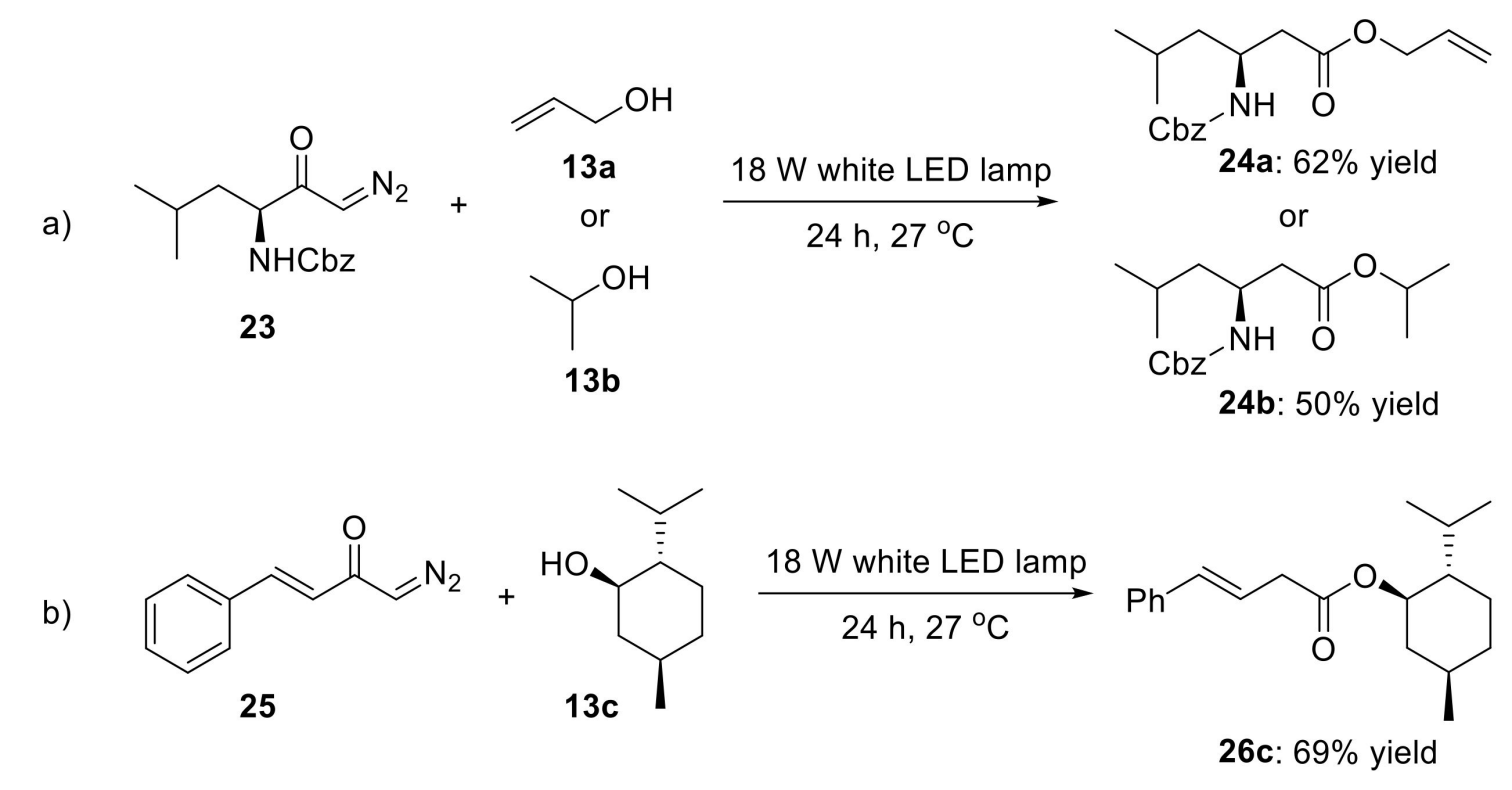
Photochemical Wolff rearrangements of either chiral diazoketones or chiral oxygen nucleophiles with commercial LED lamps.

Besides chiral α-diazoketones, the use of some readily available chiral alcohols is an alternative method to obtain enantioenriched products through photoinduced chemical Wolff rearrangement. In 2015, Burtoloso et al. demonstrated the use of commercially available LED lamps as a sustainable alternative for performing photochemical Wolff rearrangements of either chiral diazoketones **23** or chiral oxygen nucleophiles **13c** (Scheme 11) [43].

**Scheme 12:**
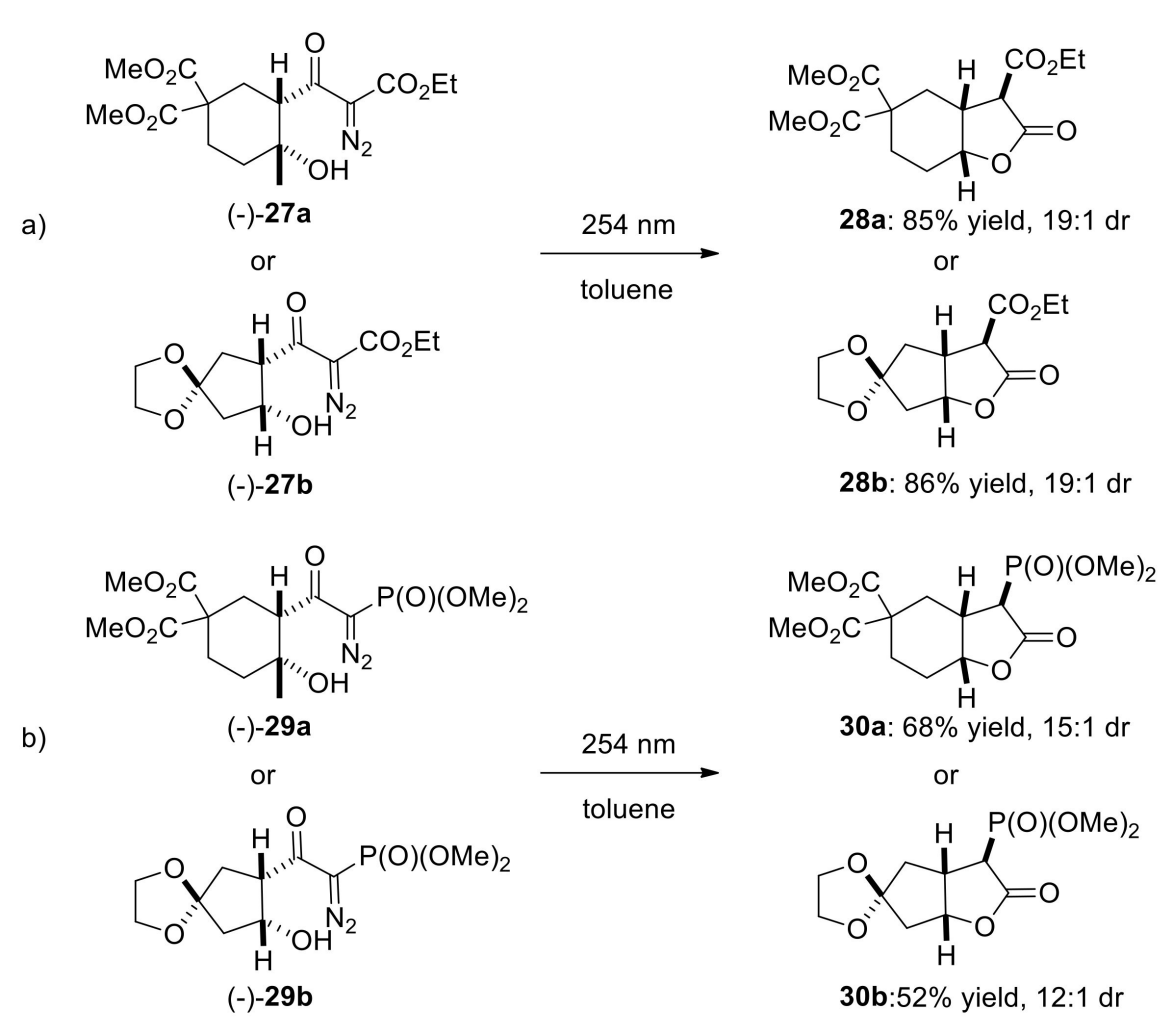
(**a**) Preparation of bicyclic and tricyclic α-carboethoxy γ-lactones via the tandem Wolff rearrangement/lactonization process; (**b**) Preparation of bicyclic and tricyclic α-dimethyl phosphonate γ-lactones via the tandem Wolff rearrangement/lactonization process.

In 2007, Zhang and Romo devised a route to bicyclic and tricyclic fused γ-lactones via a tandem Wolff rearrangement/lactonization process. The diazocarbonyl precursors were prepared by the transformation of bicyclic and tricyclic β-lactones to δ-hydroxy-α-diazo-β-ketoesters **27**. The resulting δ-hydroxy-α-diazo-β-ketoesters were then subjected to photolytic Wolff rearrangement conditions to form a ketene that was trapped efficiently by the pendant alcohol to form a γ-lactone **28** (Scheme 12a). A similar sequence of reactions could be used to convert β-lactones into phosphonate γ-lactones **30** (Scheme 12b) [44].

#### 3.1.2. Nitrogen Nucleophilic Addition

**Scheme 13:**
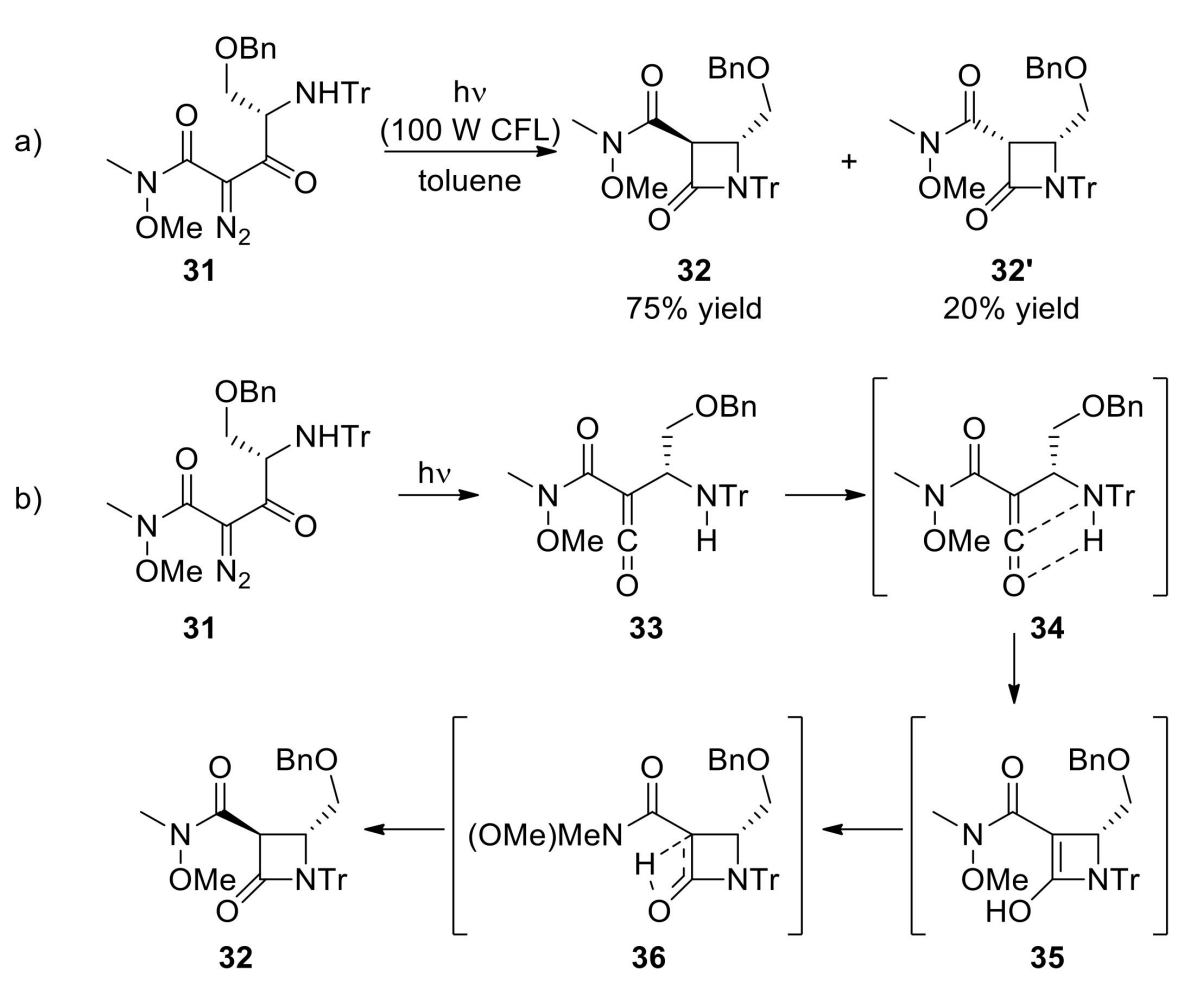
(**a**) Photolytic formation of a β-lactam via intramolecular N−H trapping of a ketene intermediate; (**b**) proposed possible mechanism. (CFL = Compact Fluorescent Light).

The combination of the Wolff rearrangement with the intramolecular addition of a nitrogen nucleophile constitutes a powerful way to prepare nitrogen-containing heterocycles. For example, it represented a key feature of the synthesis of enantiomerically pure trans-β-lactams **32** from α-amino acids (Scheme 13) [45]. Mechanistically, the Wolff rearrangement affords a ketene intermediate **33**. Based on the theoretical studies, the addition of an amine to this intermediate could occur via a cyclic transition state **34**, affording the corresponding enol amide **35**. This high-energy intermediate **35** undergoes an intramolecular tautomerization providing the final product **32** with a new chiral center at the C3 position. Since photolysis occurs in pure nonprotic solvents (toluene), it seems reasonable in this case that intramolecular tautomerization may be circumvented by impurities in the system that promote the 1,3-proton shift. Alternatively, the use of a Weinreb amide substituent allowed the control element to be located at the C3 position and the ready transformation to other central functionalities.

**Scheme 14:**
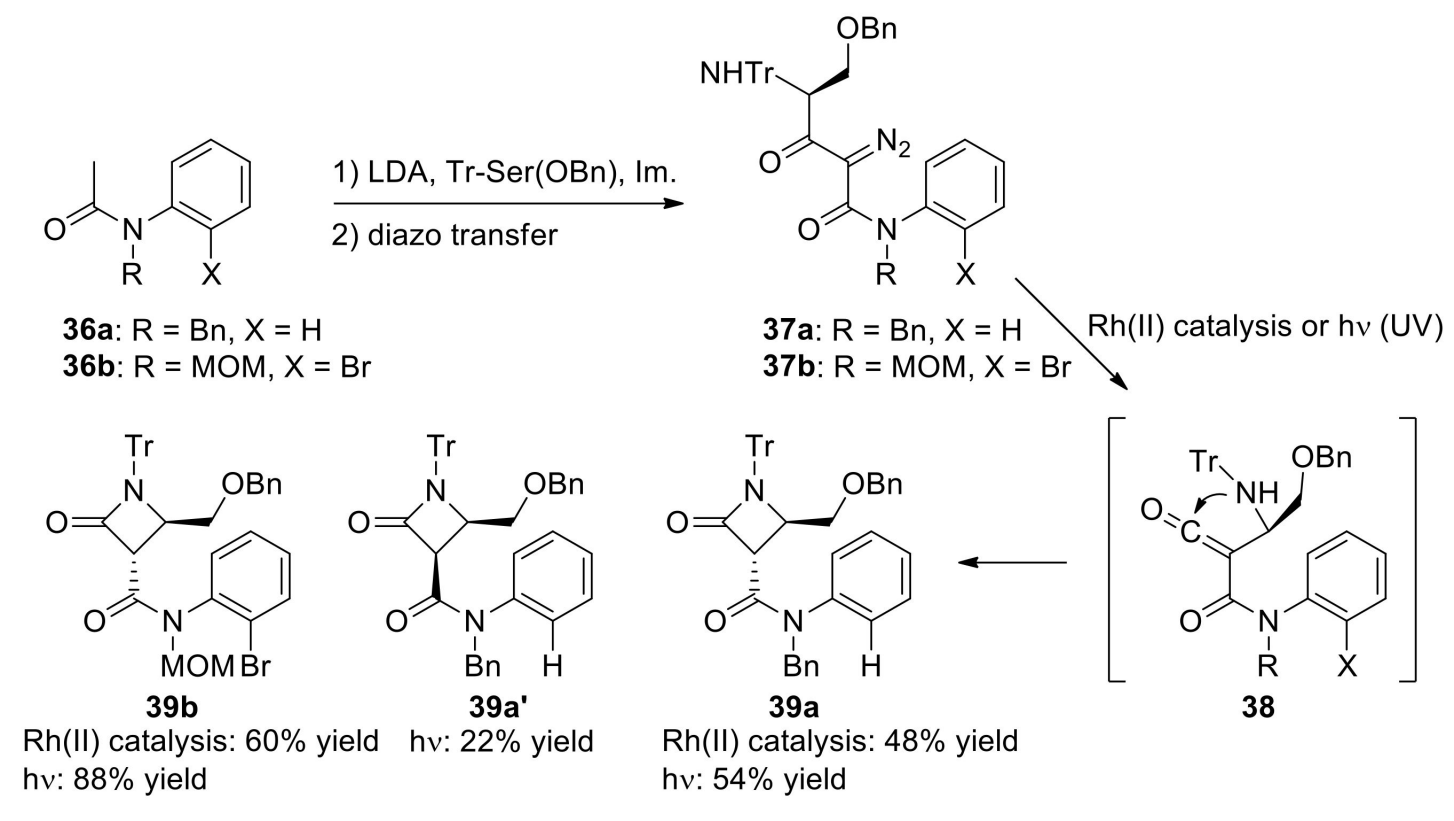
Photolytic formation of a β-lactam via intramolecular N−H trapping of a ketene under UV irradiation with a medium pressure mercury vapor lamp. (LDA: lithium diisopropylamide; Im.: Imidazole; MOM: methoxymethyl).

Another example, based on α-amino acid building block, is also from the work of the Konopelski group in the synthetic approaches to the marine natural product diazonamide A. The suitable N-protected diazo precursor **37** was prepared from a serine amino acid-based imidazolide and N-benzylacetanilide **36**. The Wolff rearrangement by photocatalysis gave better results than that via the metal catalysis (Scheme 14) [46].

**Scheme 15:**
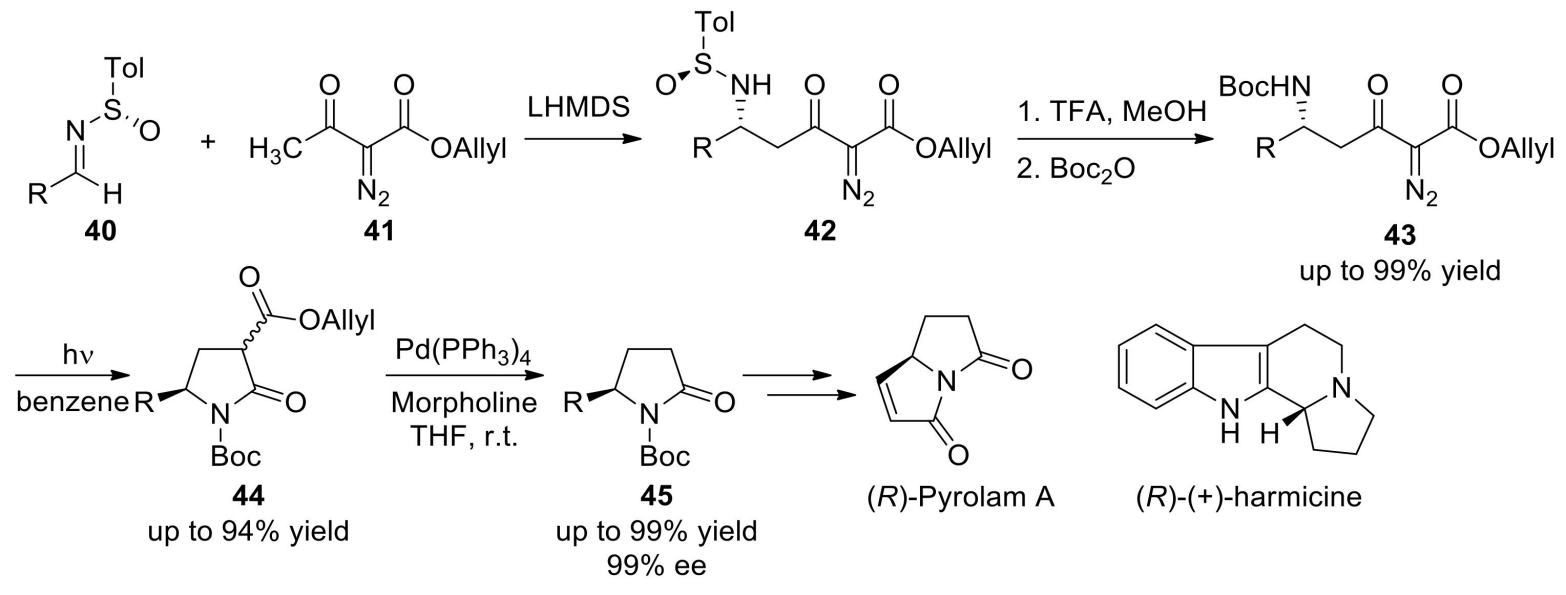
Combined Wolff rearrangement and intramolecular N−H trapping and further applications. (LHMDS: lithium hexamethyldisilazide; TFA: trifluoroacetic acid; Boc: *t*-butyloxy carbonyl).

Other examples of the intramolecular addition of nitrogen nucleophiles to oxoketenes occurred in substrates of the type that were illustrated by Wang and co-workers (Scheme 15) [47,48,49]. They devised a highly enantioselective synthesis of 2-oxo and 3-oxo pyrrolidines by diastereoselective addition of the lithium enolate of α-diazoacetoacetate **41** to chiral *N*-sulfinyl imines **40**, followed by photoinduced Wolff rearrangement [47]. The initial intention was to use chiral δ-(N-sulfinylamino)-α-diazo-β-ketoesters **42** to perform the photolytic Wolff rearrangement and thereby result in intramolecular N–H trapping leading to 2-oxopyrrolidines. However, in this case, no expected results were observed, and it was concluded that N-sulfinyl group was incompatible with the photolysis conditions. The corresponding N-Boc diazoester **43**, which was obtained by replacement of *N*-sulfinyl group by a Boc group, did produce the 2-oxo-3-allyloxycarbonyl pyrrolidine **44** upon photolysis with a high-pressure Hg lamp (λ > 300 nm). The allyloxycarbonyl group was removed by a Pd(0)-catalyzed reaction due to the poor diastereoselectivity caused by the easy epimerization at the C3 position, delivering 5-substituted 2-oxo pyrrolidines **45** in excellent yields with high enantiomeric selectivities. Later, they further expanded this methodology to the enantioselective synthesis of (*R*)-Pyrolam A [48] and (*R*)-(+)-Harmicine [49].

**Scheme 16:**
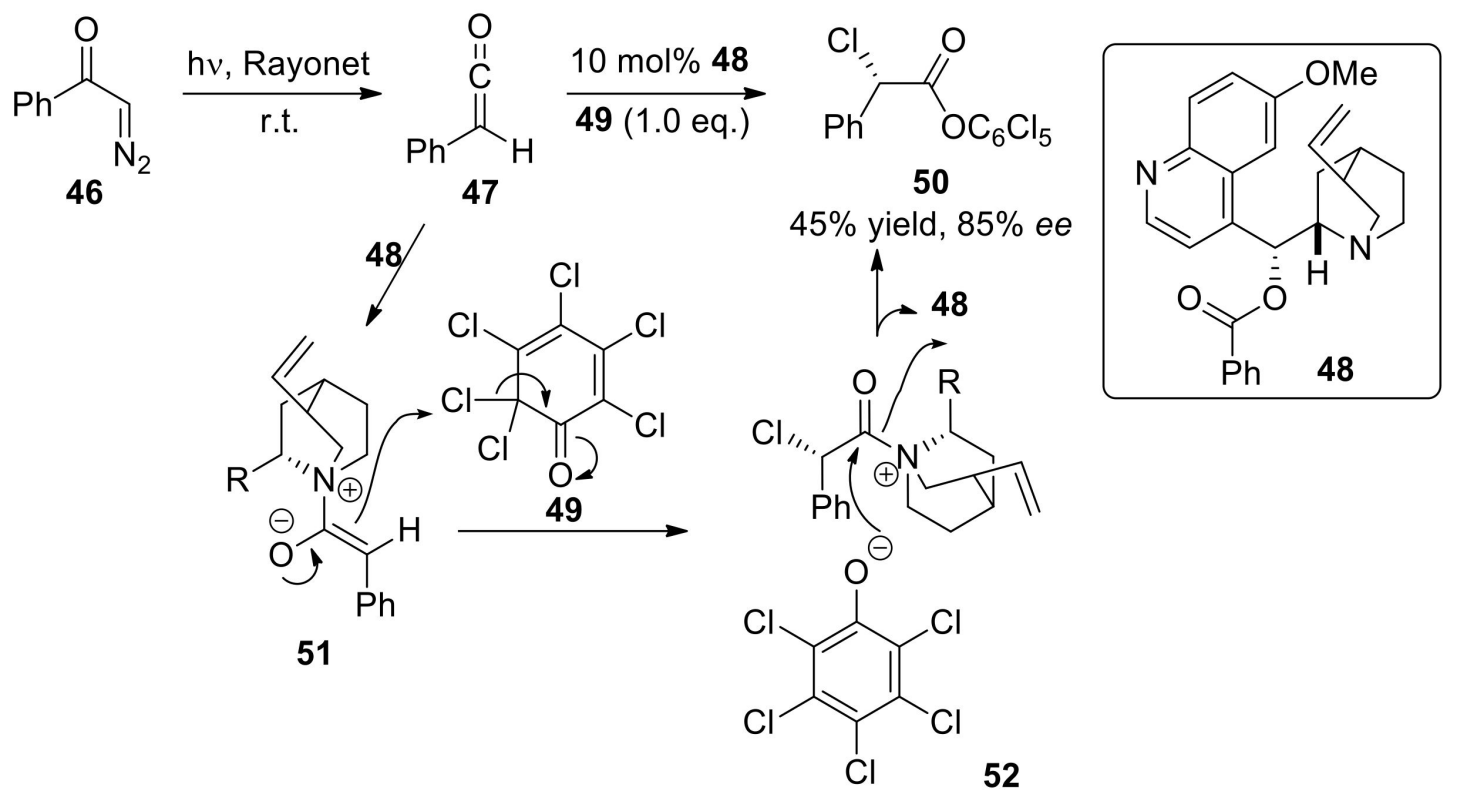
Tandem catalytic asymmetric chlorination/esterification based on Wolff rearrangement. (Rayonet photo mini-reactor: 8 × 4 W bulbs was employed for photochemical reactions.).

In 2004, Lectka and co-workers applied the generated free ketene in the photochemical Wolff rearrangement as a supplementary source for the catalytic asymmetric α-chlorination, where a cinchona alkaloid was employed as catalyst (Scheme 16) [50]. The intermolecular nucleophilic addition of benzoylquinine **48** to the in situ formed ketene in the photochemical Wolff rearrangement afforded zwitterionic enolate **51**. Tandem chlorination/esterification reaction took place by reacting with a mild electrophilic chloro source **49**. The electrophilic chloro atom would be introduced to the α-position of the enolate, producing an acylammonium salt **52** that subsequently underwent transacylation with the leaving group of the electrophile. This process produced a chiral α-chloroester **50** in 45% yield and 85% ee.

#### 3.1.3. Carbon Nucleophilic Addition

**Scheme 17:**
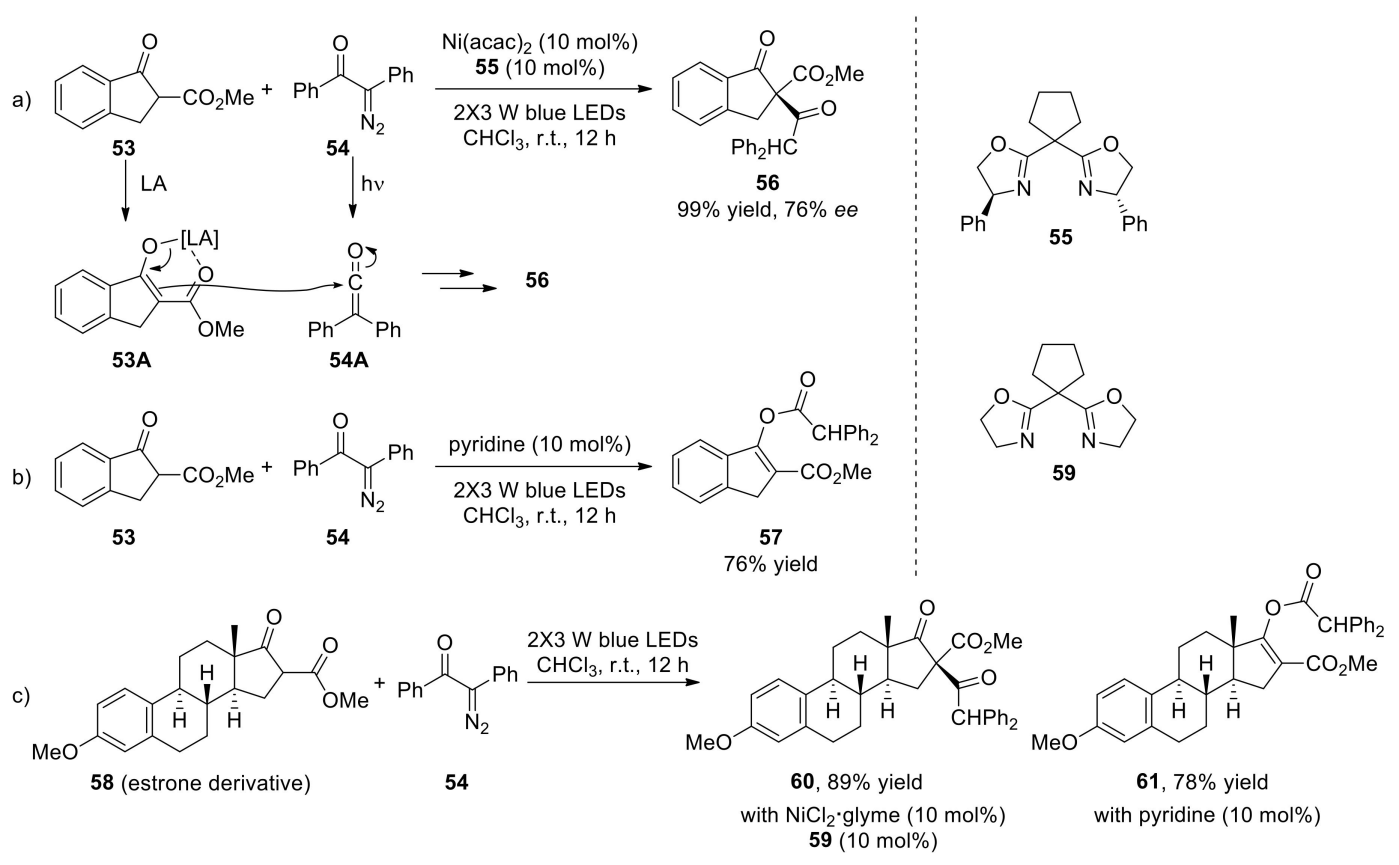
(**a**) Lewis acid-catalyzed *C*-acylation reaction of β-ketoesters with α-diazo ketones under visible-light irradiation; (**b**) Lewis base-catalyzed *O*-acylation reaction of β-ketoesters with α-diazo ketones under visible-light irradiation; (**c**) Synthetic modification.

More recently, the nucleophilic addition has been extended to carbon nucleophiles. A notable example of this protocol is *C*-acylation of β-ketoesters with α-diazo ketones by combining visible-light photoactivation with Lewis acid (LA) catalysis by Xiao, Lu, and co-workers (Scheme 17a) [51]. The success of the reaction largely depends on the visible-light-induced Wolff rearrangement and the unique catalytic activation mode, thus greatly expanding the application of ketene chemistry. A preliminary result for the asymmetric *C*-acylation reaction was given with a chiral ligand **55**. They assumed that LA-chelated enolate intermediate **53A** would react with ketene intermediate **54A** to deliver the product **56** smoothly. However, the utilization of pyridine as the Lewis base (LB) catalyst resulted in the selective *O*-acylation process, providing the enol ester product **57** (Scheme 17b). To demonstrate the synthetic utility of the method, structurally complex β-ketoester **58**, which was derived from the pharmaceutical agent estrone 3-methyl ether was subjected to the standard conditions including LA or LB catalyst. The corresponding *C*-acylation product **60** or *O*-acylation product **61** were obtained in high-yield and with complete regioselectivity (Scheme 17c).

#### 3.1.4. Nucleophilic Addition Based on Ring Contraction via In Situ Wolff Rearrangement

Ring contraction in ring systems has been widely used as one of the most effective methods to produce high strained molecules. In this respect, a major advantage of Wolff rearrangement is its independence of ring-opening/ring-closure sequences. There are a large number of examples that show that it can change the size of the ring, rather than make the ring with an acyclic precursor. Photolysis is a good choice for ring contraction. An illustrative few examples of ring contractions in total synthesis are selected to demonstrate the potential use for different types of rings [52,53,54,55,56].

**Scheme 18:**
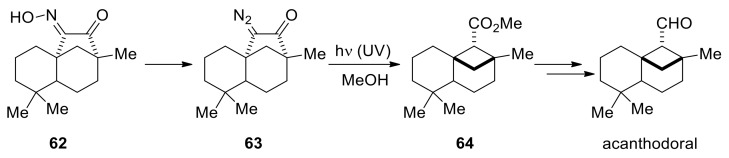
Wolff rearrangement for the synthesis of acanthodoral.

In 2004, Zhang and Koreeda proved the efficacy of the Wolff rearrangement in strained ring synthesis for the marine antibiotic acanthodian. It is a sesquiterpene aldehyde possessing a bridged bicyclo[3.2.1]heptane. The construction of this highly-strained bicyclo[3.1.1]heptane skeleton was realized by employing a Wolff rearrangement of the corresponding α-diazoketone **63** with the bicyclo[3.2.1]octane system (Scheme 18) [52].

**Scheme 19:**
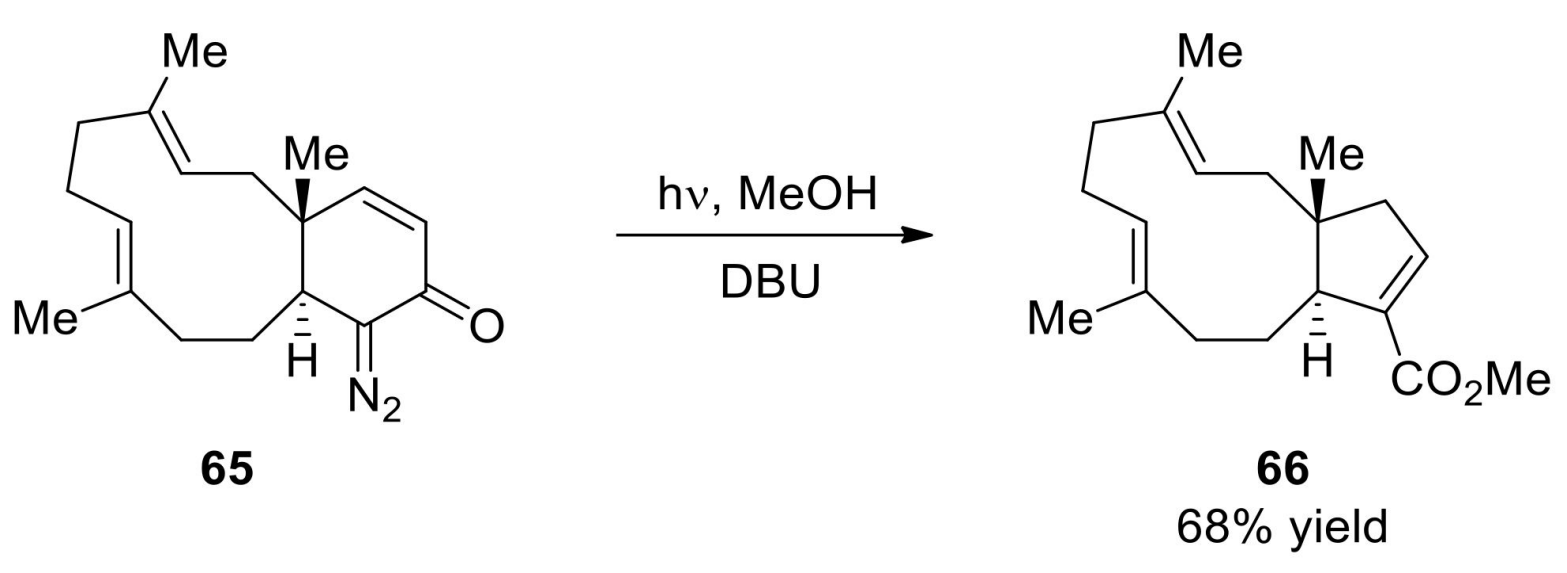
Wolff rearrangement of a diazocyclohexenone derivative. (A photoreactor equipped with a 450 W Hanovia UV lamp: medium pressure mercury lamp and a Pyrex cooling jacket were used for irradiation.).

In 2006, the Corey group achieved a concise and stereoselective total synthesis of the dolabellane-type marine natural products with bicyclic structures via an efficient synthetic strategy which required a final ring contraction. In this synthesis, photoirradiation of the advanced intermediate **65** in MeOH followed by solvent removal and thermal treatment with 1,8-diazabicyclo[5.4.0]undec-7-ene (DBU) generated the required ring-contracted ester **66** in 68% yield (Scheme 19) [53]. The success of this unique and highly efficient ring-contraction process based on the photochemical Wolff rearrangement, given the number of potential side reactions of an intermediate carbene, generated so close to a neighboring olefinic linkage and ring junction deserved to be mentioned.

**Scheme 20:**
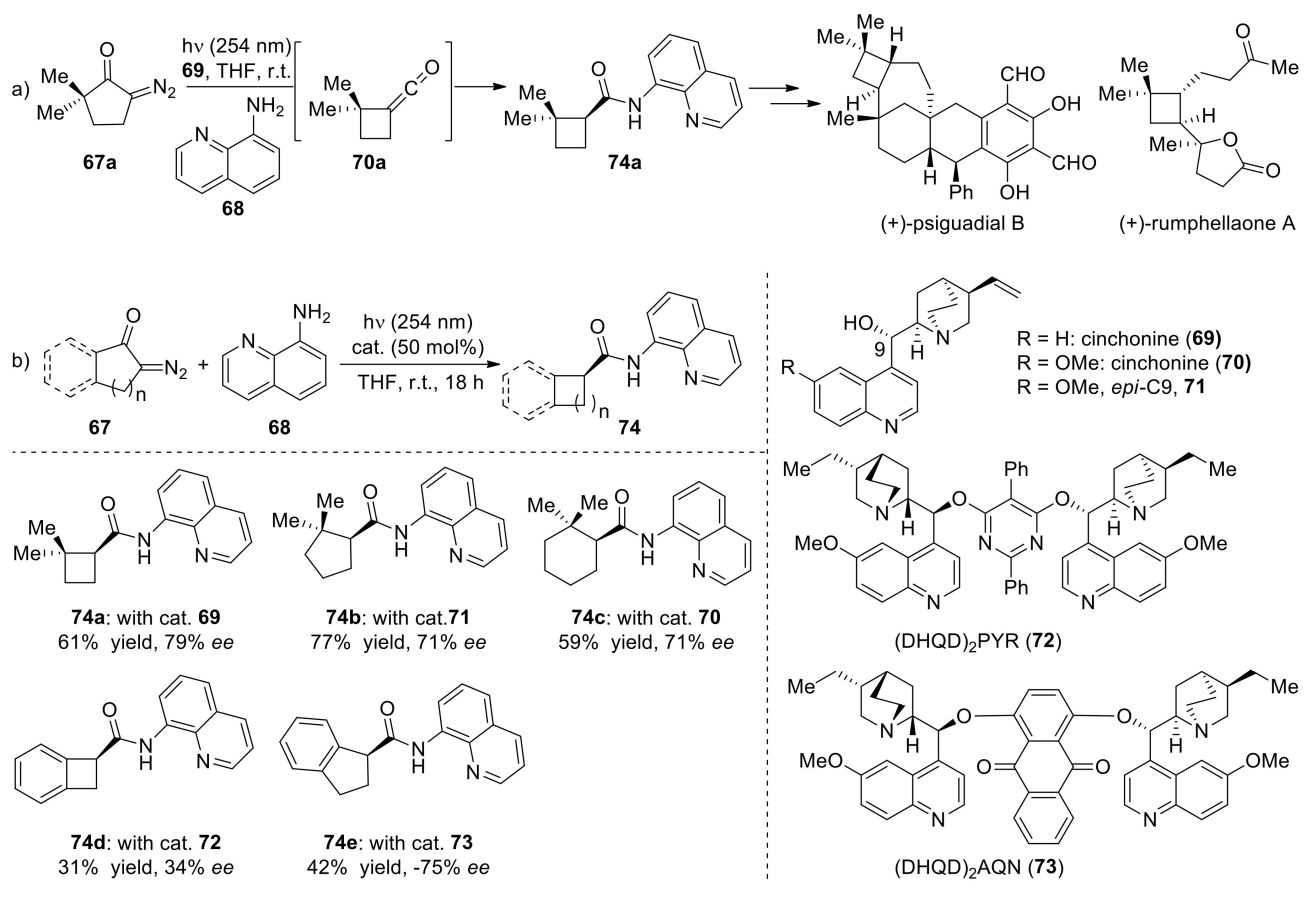
(**a**) Enantioselective synthesis of natural products via tandem Wolff rearrangement/asymmetric ketene addition strategy; (**b**) the evaluation of this strategy.

The structural motifs of cyclobutane exist in a variety of natural products and pharmaceuticals. 1,2-Cyclobutanes are also versatile synthetic intermediates, as the inherent ring strain of these structures engenders them with unique reactivity that can be leveraged in various transformations to construct complex frameworks. Reisman and co-workers reported the enantioselective synthesis of trans-cyclobutane-containing natural products, such as (+)-Psiguadial B [54,55] and (+)-rumphellaone A (Scheme 20a) [56]. The synthetic strategies were both started with the de novo construction of the trans-fused cyclobutane ring via a tandem Wolff rearrangement/asymmetric ketene addition. They further applied this tandem reaction to other α-diazoketone substrates with different ring size and evaluated selected cinchona derivatives **69-73** (Scheme 20b) [55]. Unfortunately, at present, there is no general catalyst for Wolff rearrangement/enantioselective addition of 8-aminoquinoline **68**, which needs further mechanical studies to provide a basis for improving the generality of this reaction in the future. 

### 3.2. Cycloaddition Reactions Based on the In Situ Wolff Rearrangement

#### 3.2.1. Formal [2 + 2] Cycloaddition Reaction

**Scheme 21:**
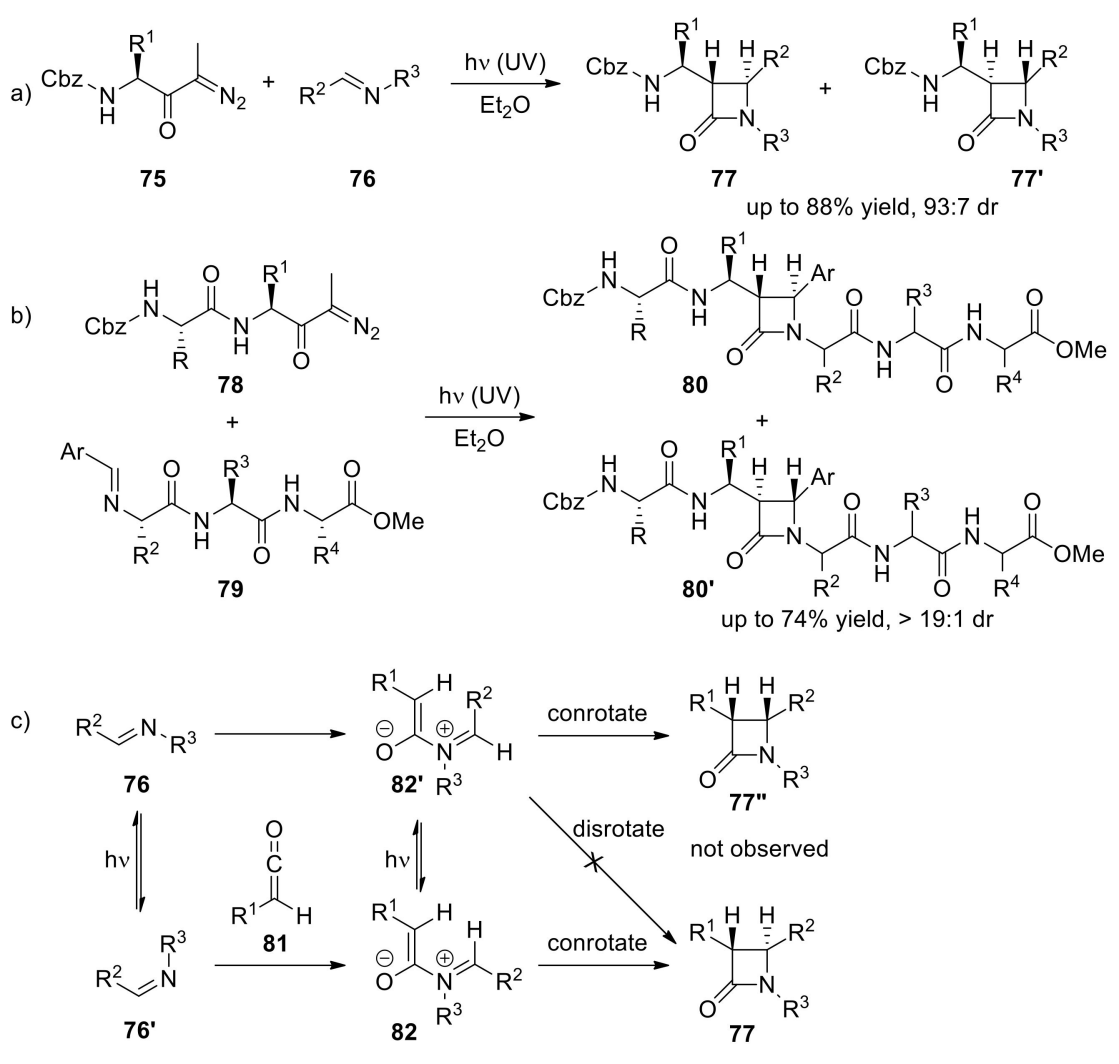
(**a**) Photoinduced β-lactam formation via a formal [2 + 2] cycloaddition of ketene from amino acid-derived diazoketone to imine; (**b**) Photoinduced β-lactam formation via a formal [2 + 2] cycloaddition from peptidic starting materials; (**c**) proposed mechanism.

The Podlech group presented a formal [2 + 2] cycloaddition reaction (the Staudinger synthesis) of photochemically generated ketenes from amino acid-derived diazoketones as precursors and aromatic imines, exclusively furnishing trans-β-lactam **77** as a mixture of two isomers with good yield and diastereoselectivity (Scheme 21a) [57,58,59,60]. They further disclosed a useful modification of this β-lactam synthesis in which peptidic starting materials were used instead. Especially noteworthy is the introduction of additional stereogenic centers on the imines, giving rise to the corresponding products with up to > 19:1 dr (Scheme 21b) [61]. On the basis of the experimental results and mechanistic studies, it is assumed that the selective formation of trans diastereoisomers results from the ability of imines **76** and acyclic adducts **82**, **82′** to isomerize upon UV irradiation (Scheme 21c) [62].

#### 3.2.2. Formal [4 + 2] Cycloaddition Reaction

**Scheme 22:**
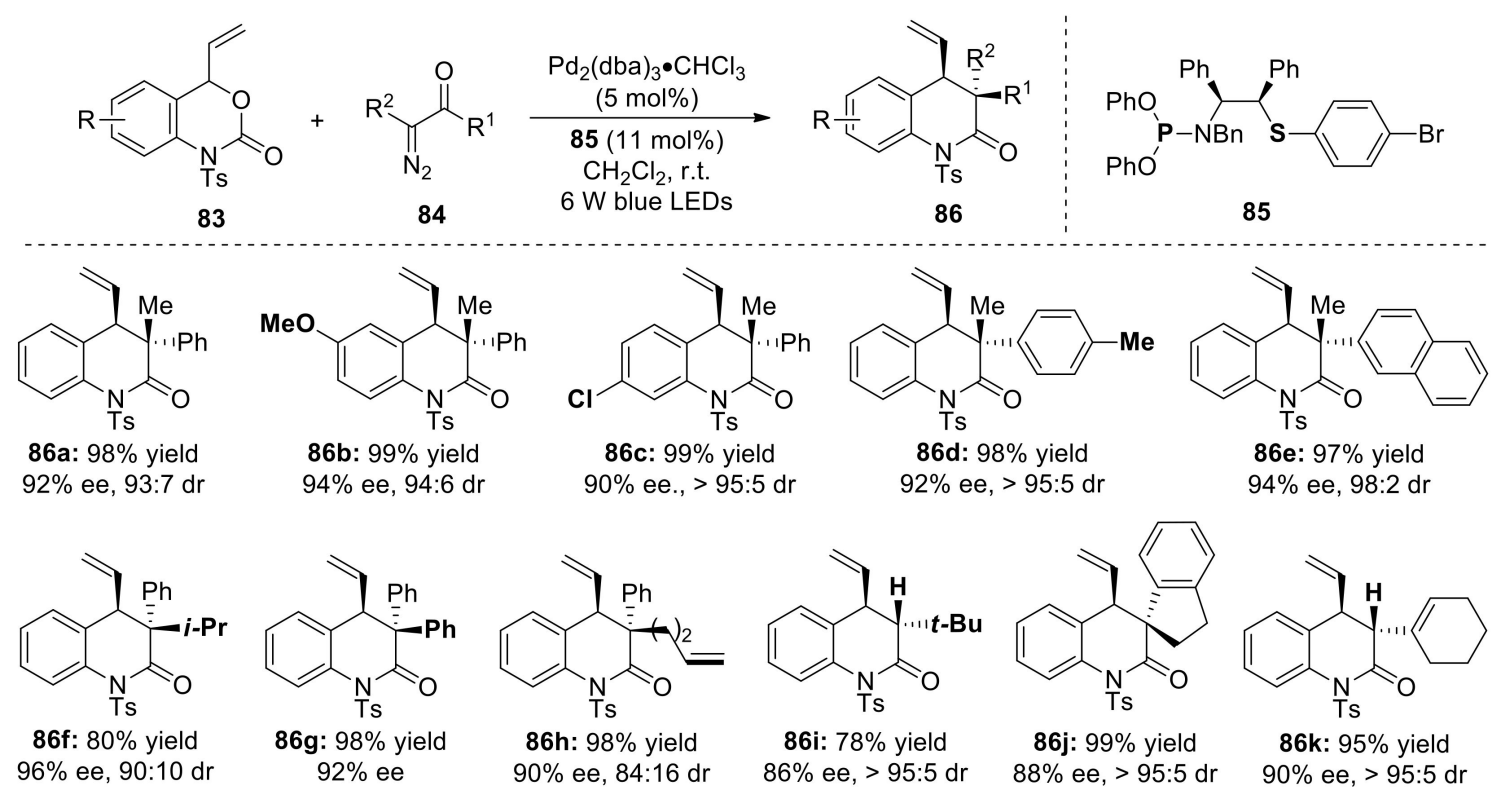
Visible-light-induced, Pd-catalyzed asymmetric [4 + 2] cycloadditions of vinyl benzoxazinones with α-diazoketones.

**Scheme 23:**
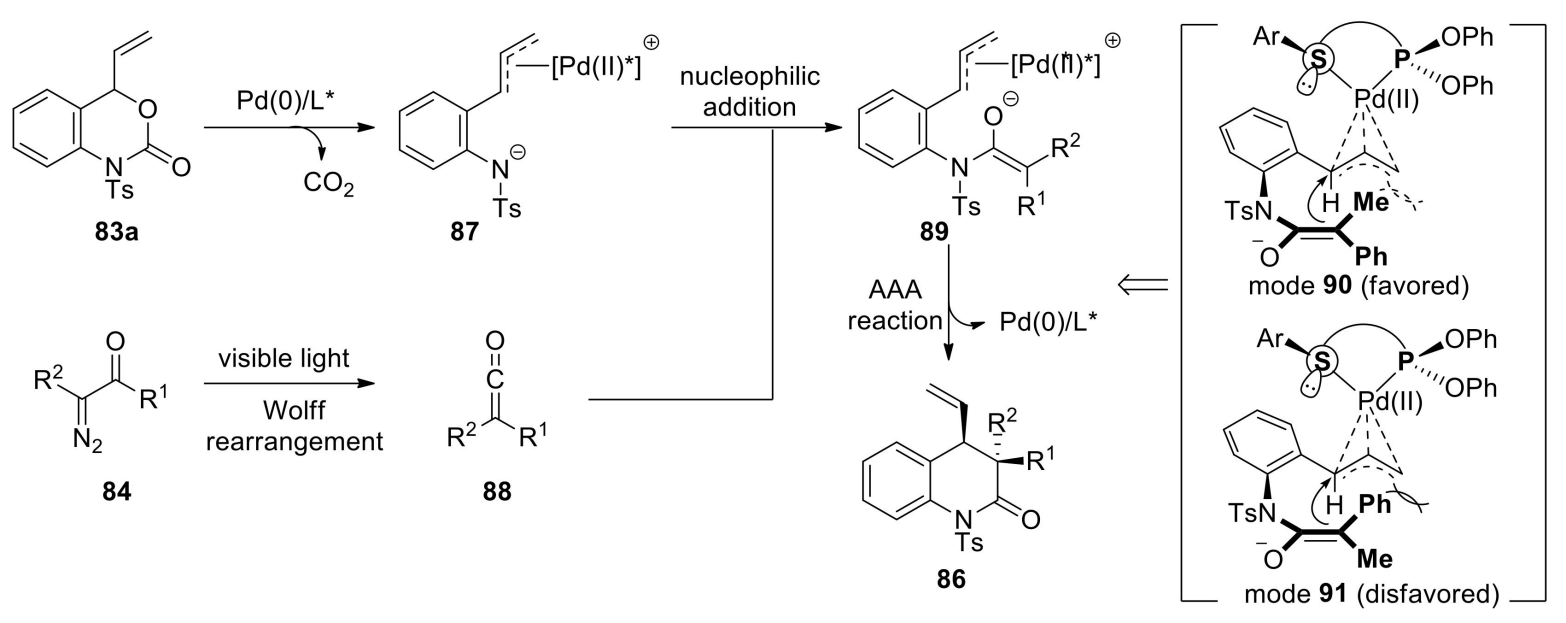
A proposed mechanism for Pd-catalyzed asymmetric [4 + 2] cycloadditions of vinyl benzoxazinones with α-diazoketones.

In 2017, the group of Lu and Xiao demonstrated a formal [4 + 2] cycloaddition reaction of vinyl benzoxazinones **83** with α-diazoketones **84** via sequential visible-light photoactivation and palladium catalysis with their P,S-hybrid ligands [63]. A variety of chiral quinolinones **86** were produced with high reaction efficiency and stereocontrol in this transformation (Scheme 22). This study features the substantial practicality of the visible-light-driven photoconversion of α-diazoketones and the unique reactivity of the resulting ketenes in Pd-catalyzed asymmetric cycloadditions [64,65]. As depicted in the proposed reaction pathway for the above process, Pd-containing 1,4-dipole **87** was generated in situ by decarboxylative process of vinyl benzoxazinone **83a** with a chiral Pd(0) catalyst; meanwhile, reactive ketene intermediate **88** was formed via the visible-light-induced Wolff rearrangement of α-diazoketone **84**. Then, final product **86** was produced after a sequential intermolecular nucleophilic addition/intramolecular asymmetric allylic alkylation (AAA) process. Furthermore, the possible stereocontrol modes were proposed to rationalize the asymmetric induction: **90** seems more favored than **91** due to less steric repulsion between the methyl group and the chiral π-allyl-Pd fragment. In this work, many experiments were performed to elucidate the high reaction selectivity because the α-diazoketones and ketenes are unstable to Pd(0/II) catalysts. It is noteworthy that chiral P,S ligands are not only important for good enantiocontrol, but also for reducing the catalytic decomposition of α-diazoketone (Scheme 23).

#### 3.2.3. Formal [5 + 2] Cycloaddition Reaction

**Scheme 24:**
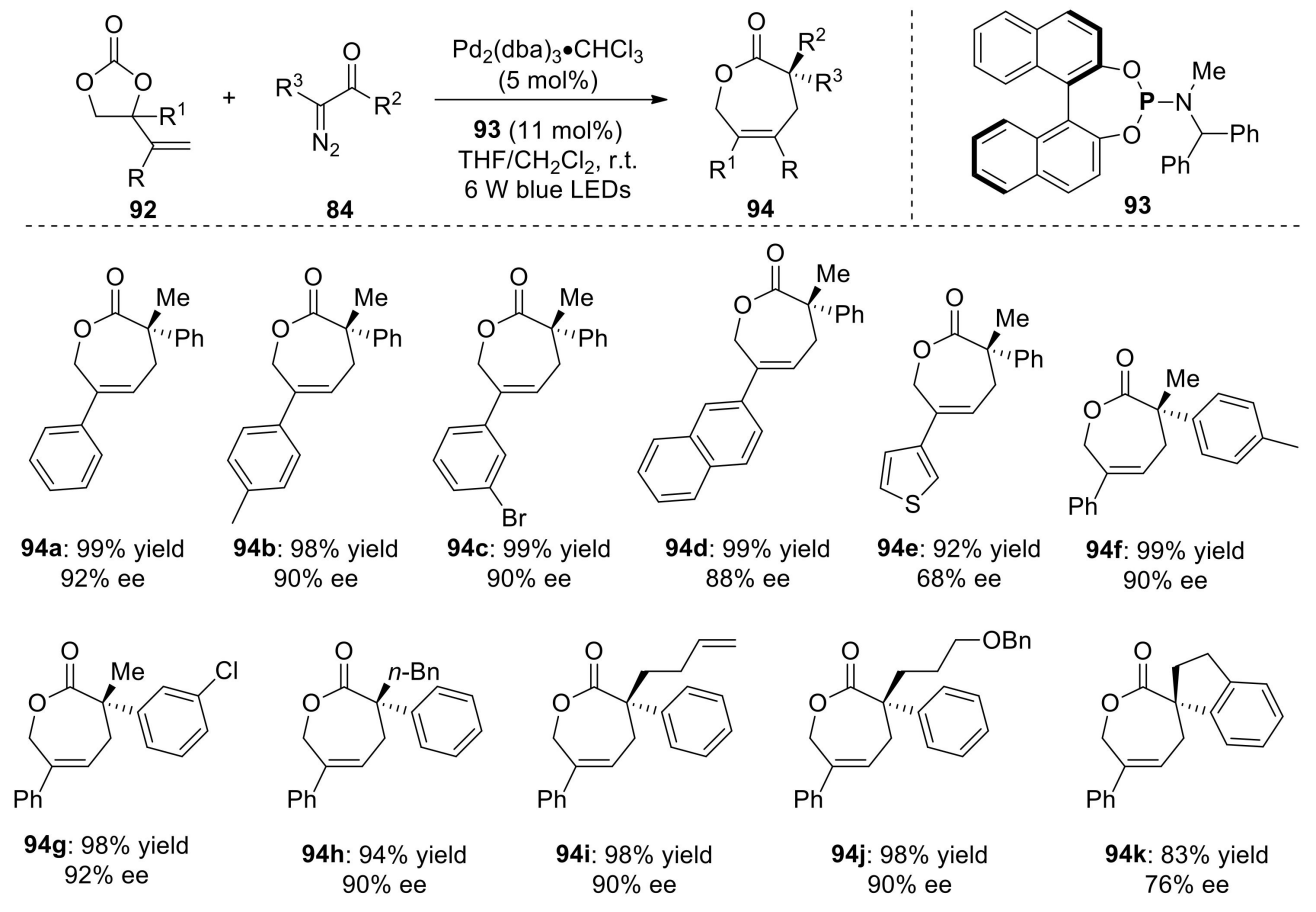
Visible-light-induced, Pd-catalyzed asymmetric [5 + 2] cycloaddition of vinylethylene carbonates with α-diazoketones.

**Scheme 25:**
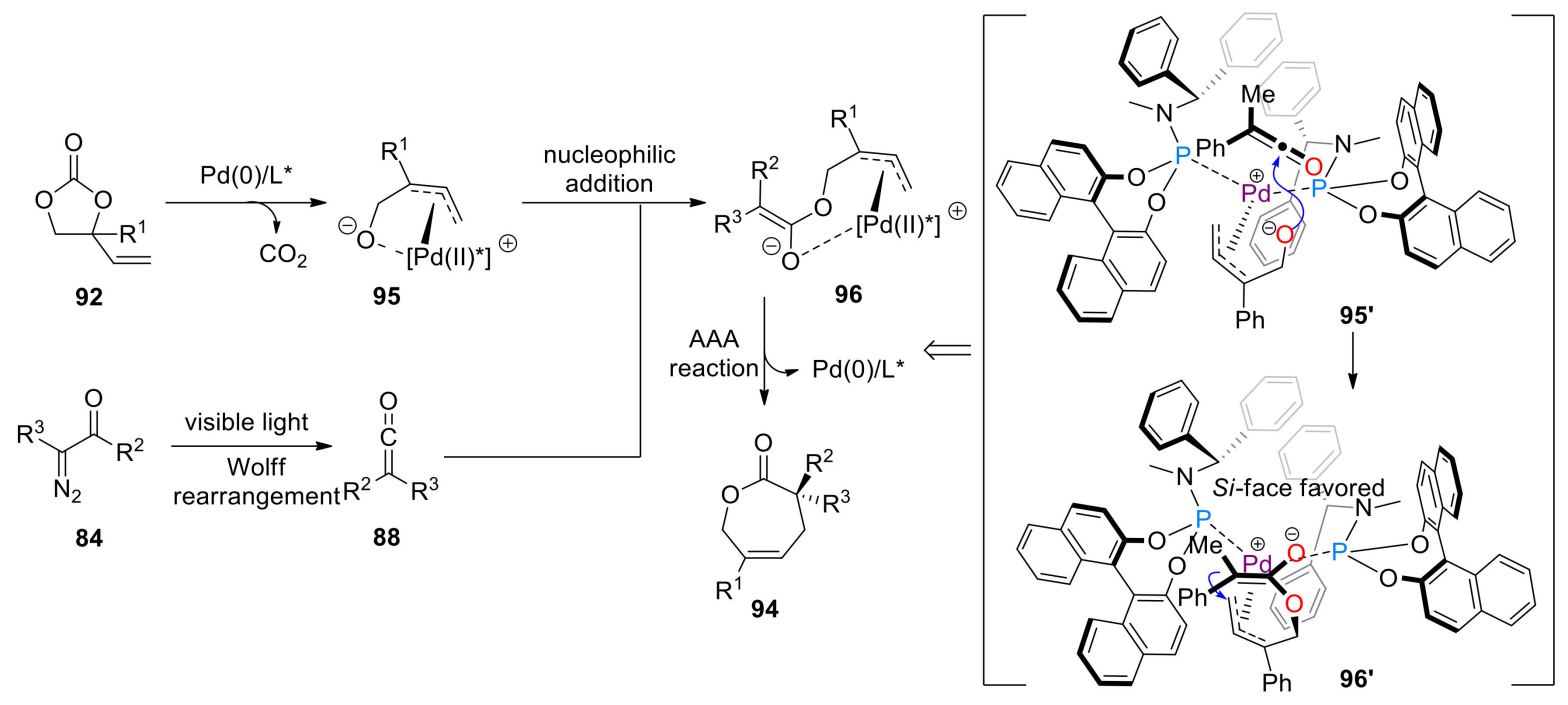
A general mechanism for the [5 + 2] cycloaddition of vinylethylene carbonates with α-diazoketones.

In 2019, the same group successfully achieved the first visible-light-induced, Pd-catalyzed asymmetric [5 + 2] cycloaddition of vinylethylene carbonates **92** with α-diazoketones **84** [66]. This methodology provides an enantioselective approach for accessing a variety of 7-membered lactones **94** bearing chiral quaternary stereocenters with good reaction efficiency and high enantioselectivity (up to 99% yield, 92% ee) (Scheme 24). The general reaction mechanism of vinylethylene carbonates **92** with α-diazoketones **84** is illustrated. Initially, decarboxylation of vinylethylene carbonate in the presence of a Pd(0) catalyst generates Pd-containing π−allyl dipole **95**. Simultaneously, visible- light irradiation of α-diazoketones **84** would tracelessly release ketene species **88** via a Wolff rearrangement. Then nucleophilic addition of the oxygen anion of the intermediate to ketenes could smoothly provide a new zwitterionic intermediate **96** followed by an intramolecular AAA reaction of the enolate fragment, and π−allyl-Pd fragment would furnish the desired seven-membered lactones **94**. Based on the mechanistic studies, two possible modes were proposed to explain the stereochemical outcome, which suggested that the binding of two phosphoramidite ligands to the palladium center was responsible for the high reaction efficiency and enantioselectivity (Scheme 25).

#### 3.2.4. Formal [3 + 2] Cycloaddition Reaction

**Scheme 26:**
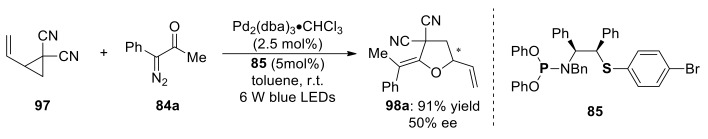
Visible-light-induced, Pd-catalyzed [3 + 2] cycloaddition reactions of vinyl cyclopropanes and α-diazoketones.

Soon after, the same group also extend the strategy that combines visible light photoactivation and palladium catalysis to [3 + 2] cycloaddition reactions of vinyl cyclopropanes **97** and α-diazoketones **84**. After condition optimizations, the desired tetrahydrofuran product **98a** was obtained with high yield and moderate enantioselectivity (Scheme 26) [67].

## 4. Asymmetric Alkylation

**Scheme 27:**
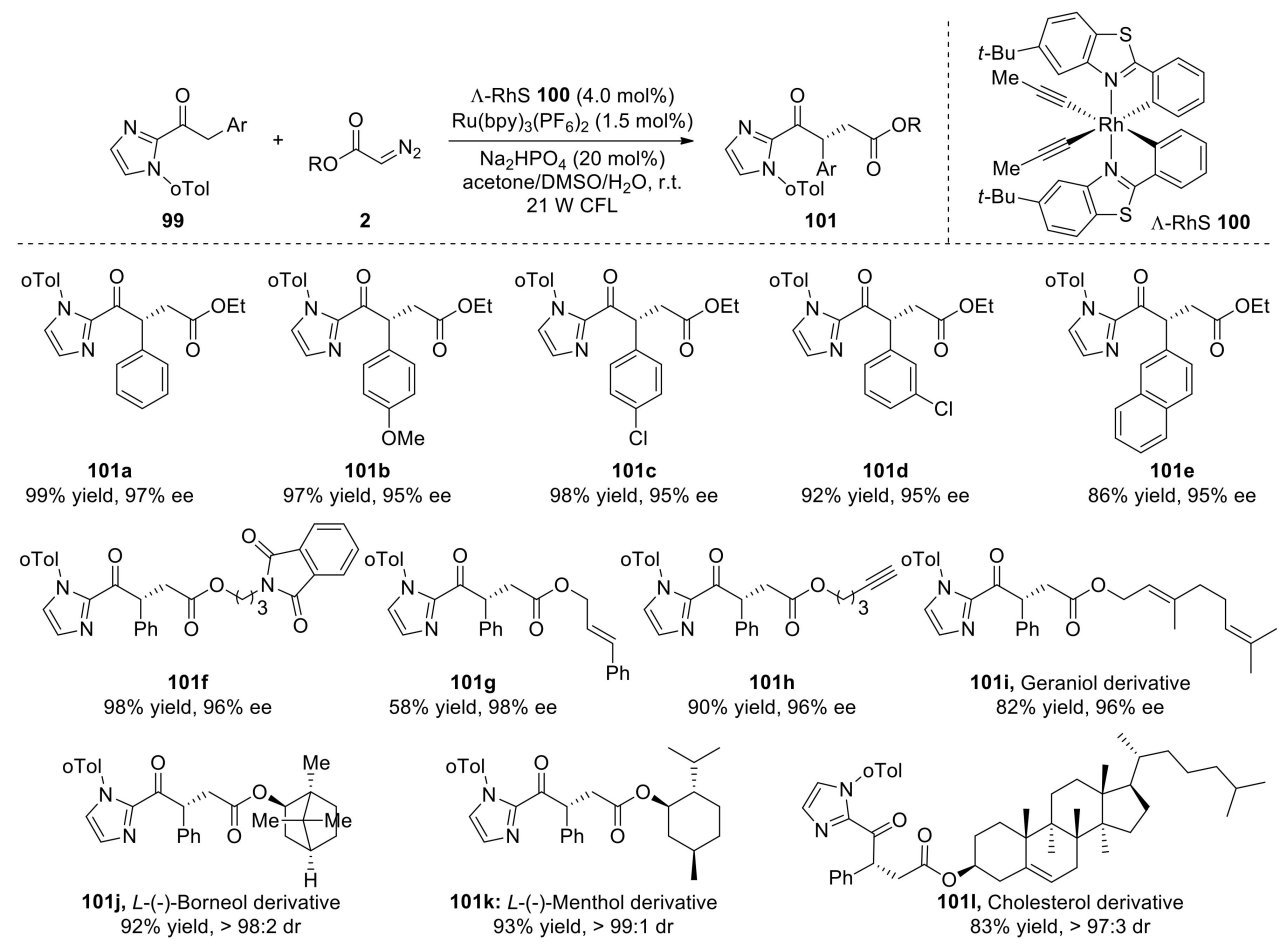
Visible-light-activated asymmetric α-alkylation of 2-acyl imidazoles catalyzed by a chiral-at-metal rhodium-based Lewis acid in combination with a photoredox sensitizer. (DMSO: dimethyl sulfoxide).

**Scheme 28:**
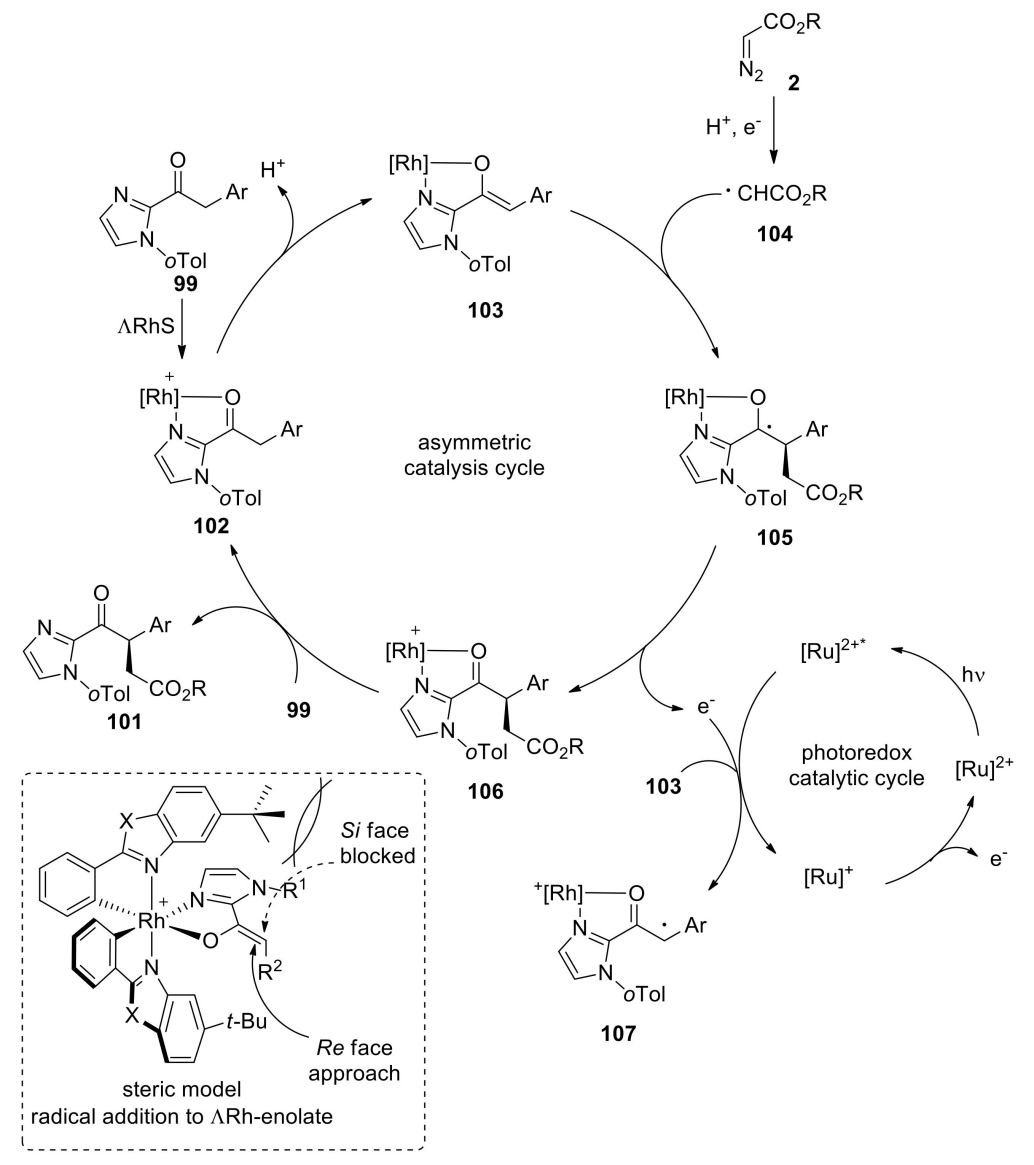
Proposed mechanism and steric model for the visible-light-induced rhodium-catalyzed asymmetric radical addition to enolates.

In 2016, Meggers et al. reported that α-diazo carboxylic esters **2** were suitable for visible-light-activated asymmetric α-alkylation of 2-acyl imidazoles **99** catalyzed by a chiral-at-metal rhodium-based Lewis acid in combination with a photoredox sensitizer. This novel proton- and redox-neutral method provided excellent yield (up to 99%) and enantioselectivity (up to >99% ee) of respective 1,4-dicarbonyl compounds **101** with a broad functional group compatibility (Scheme 27) [68]. Mechanistically, substrate coordination with rhodium complex and base-induced deprotonation initially delivered the key rhodium enolate intermediate **103**, which acted as a single electron donor for a photo-excited ruthenium complex. Simultaneously, one-electron transfer from the in situ generated [Ru(bpy)_3_^I^] to α-diazo ester **2** formed an electron-deficient radical **104**. The addition of this radical to the electron-rich enolate followed by single electron transfer (SET) oxidation leads to a product-coordinated rhodium intermediate **106**. Then, the product is released, and the recoordination of the new substrate with the metal complex completes the whole catalytic cycle. Experimental studies support the enhancing visible-light absorption and oxidation potential decreasing of the key rhodium enolate intermediate. Regarding the stereodetermining step, the asymmetric induction occurs in the course of the radical addition to the enolate. X-ray crystal diffraction shows that the *Si* face of the prochiral C(sp^2^) of the enolate is sterically hindered by the tert-butyl groups of the C2 symmetric Λ-RhS (Scheme 28). A computational study in collaboration with the Houk group further suggested that distortion of the rhodium-bound enolate and its cyclo metalating ligand skeleton is the controlling factor in enforcing enantioselectivity [69].

## 5. Conclusions and Prospects

In conclusion, with the numerous recent advances in the field of photocatalysis, diazo compounds have proven to be amongst the most versatile reagents. Among the reported examples, asymmetric reactions based on the in situ Wolff rearrangement were the most widely studied area where ketenes function as reaction intermediates. In this regard, selective bioconjugation in proteins and nucleic acids and carbohydrates exploiting diazocarbonyl reactivity is obvious, which provides a new tool for drug discovery. Furthermore, visible-light-induced enantioselective transformations of diazo compounds have also demonstrated new significant areas recently. It goes without saying that this research field has experienced a revival of interest in photoinitiated reactions, and will continue to be flourishing in the future due to the increased understanding of modern photochemistry. Despite these great achievements, the enantioselective reactions of diazo compounds under photo-irradiation still remain some challenges. First, the most successful asymmetric reactions are greatly limited to stabilized diazo compounds. Second, the limited catalysis systems restrict their applications in diversity-directed organic synthesis. Last but not least, the radical reactions are typically difficult due to the control of reactivity and selectivity for radical chemistry. In summary, further studies on the substrate scope, catalysis system, and diversity of reactions are still highly desirable. We hope that this review will be a useful reference and inspiration to stimulate more interest and further development in this interesting and emerging field.
